# Efficient differentiation of human embryonic stem cells to arterial and venous endothelial cells under feeder- and serum-free conditions

**DOI:** 10.1186/s13287-015-0260-5

**Published:** 2015-12-30

**Authors:** Gopu Sriram, Jia Yong Tan, Intekhab Islam, Abdul Jalil Rufaihah, Tong Cao

**Affiliations:** Oral Sciences Disciplines, Faculty of Dentistry, National University of Singapore, Singapore, 119083 Singapore; Institute of Medical Biology, Agency for Science, Technology and Research (A*STAR), 8A Biomedical Groove, #06-06 Immunos, Singapore, 138648 Singapore; Oral and Maxillofacial Surgery Disciplines, Faculty of Dentistry, National University of Singapore, Singapore, 119083 Singapore; Cardiac, Thoracic and Vascular Surgery (CTVS) Laboratory, Department of Surgery, Yong Loo Lin School of Medicine, National University of Singapore, Singapore, 117510 Singapore; Singapore-Technion Alliance For Research and Technology (START) Regenerative Medicine Laboratory, Campus for Research Excellence And Technological Enterprise (CREATE), Singapore, 138602 Singapore; NUS Graduate School for Integrative Science and Engineering, Singapore, 117456 Singapore; Tissue Engineering Program, Life Sciences Institute, National University of Singapore, Singapore, 117456 Singapore

**Keywords:** Human embryonic stem cells, Endothelial differentiation, Arterial, Venous, Serum-free, Feeder-free

## Abstract

**Background:**

Heterogeneity of endothelial cells (ECs) is a hallmark of the vascular system which may impact the development and management of vascular disorders. Despite the tremendous progress in differentiation of human embryonic stem cells (hESCs) towards endothelial lineage, differentiation into arterial and venous endothelial phenotypes remains elusive. Additionally, current differentiation strategies are hampered by inefficiency, lack of reproducibility, and use of animal-derived products.

**Methods:**

To direct the differentiation of hESCs to endothelial subtypes, H1- and H9-hESCs were seeded on human plasma fibronectin and differentiated under chemically defined conditions by sequential modulation of glycogen synthase kinase-3 (GSK-3), basic fibroblast growth factor (bFGF), bone morphogenetic protein 4 (BMP4) and vascular endothelial growth factor (VEGF) signaling pathways for 5 days. Following the initial differentiation, the endothelial progenitor cells (CD34^+^CD31^+^ cells) were sorted and terminally differentiated under serum-free conditions to arterial and venous ECs. The transcriptome and secretome profiles of the two distinct populations of hESC-derived arterial and venous ECs were characterized. Furthermore, the safety and functionality of these cells upon in vivo transplantation were characterized.

**Results:**

Sequential modulation of hESCs with GSK-3 inhibitor, bFGF, BMP4 and VEGF resulted in stages reminiscent of primitive streak, early mesoderm/lateral plate mesoderm, and endothelial progenitors under feeder- and serum-free conditions. Furthermore, these endothelial progenitors demonstrated differentiation potential to almost pure populations of arterial and venous endothelial phenotypes under serum-free conditions. Specifically, the endothelial progenitors differentiated to venous ECs in the absence of VEGF, and to arterial phenotype under low concentrations of VEGF. Additionally, these hESC-derived arterial and venous ECs showed distinct molecular and functional profiles in vitro. Furthermore, these hESC-derived arterial and venous ECs were nontumorigenic and were functional in terms of forming perfused microvascular channels upon subcutaneous implantation in the mouse.

**Conclusions:**

We report a simple, rapid, and efficient protocol for directed differentiation of hESCs into endothelial progenitor cells capable of differentiation to arterial and venous ECs under feeder-free and serum-free conditions. This could offer a human platform to study arterial–venous specification for various applications related to drug discovery, disease modeling and regenerative medicine in the future.

**Electronic supplementary material:**

The online version of this article (doi:10.1186/s13287-015-0260-5) contains supplementary material, which is available to authorized users.

## Background

The vascular system consists of a complex network of arteries and veins that are lined by a monolayer of cells called endothelial cells (ECs). Although the arterial and venous ECs share certain common molecular signatures such as the expression of pan-endothelial markers (CD31, vascular endothelial cadherin (VE-CAD), and von Willebrand factor (vWF)), they do possess certain distinct molecular profiles [1, 2]. Such molecular distinction seems to occur quite early in the development even before the onset of blood flow and involves the interplay of various signaling pathways such as sonic hedgehog (Shh), vascular endothelial growth factor (VEGF), Notch, cyclic adenosine monophosphate (cAMP), and chicken ovalbumin upstream promoter-transcription factor II (COUP-TFII) [3]. Arterial ECs are characterized by expression of Ephrin-B2, Delta-like 4 (DLL4), Hey-1, Hey-2, neuropilin-1 (NRP1), Notch-1, Notch-4, chemokine receptor-4 (CXCR4), Jag-1 and Jag-2, while the venous ECs express Eph-B4, Lefty-1, Lefty-2, neuropilin-2 (NRP2) and COUP-TFII [1]. Due to lack of access to human embryos, most of our understanding regarding the molecular mechanisms of arterial–venous specification is based on studies in zebrafish, *Xenopus* and mouse embryos, and a few studies using stem/progenitor cells. Additionally, genetic, molecular and functional studies of human ECs are limited by the availability of umbilical, neonatal or adult sources.

Recent advances in stem cell biology have provided a surrogate tool to study human development through pluripotent stem cells (PSCs) that include human embryonic stem cells (hESCs) and induced pluripotent stem cells (iPSCs) [4]. Differentiation of PSCs into ECs is of growing interest as it provides an opportunity to study vascular development in both physiological and diseased states. Secondly, the PSC-derived ECs could serve as a surrogate human vascular model to study various cellular and molecular aspects of angiogenesis [5]. Furthermore, these cells also provide access to abundant populations of cells for the pharmaceutical industry to screen and develop novel cardiovascular compounds [6, 7]. Finally, in the long term, these cells have the potential for cellular therapy to repair ischemic tissues and develop tissue-engineered vascular grafts.

We and others have reported differentiation of hPSCs towards mature and functional ECs [8–17]. Briefly, these protocols involve: (1) embryoid body-based differentiation, (2) co-culture of PSCs over murine stromal cells, and (3) monolayer differentiation over extracellular matrix (ECM) proteins like Matrigel and collagen IV [5, 18]. Despite the tremendous progress in differentiation of hESCs towards endothelial lineage, very limited data are available on how these stem cells could be coaxed into arterial or venous ECs. Secondly, these differentiation protocols have limitations such as low differentiation efficiency and use of xenogeneic (animal-derived) products such as fetal bovine serum (FBS), murine feeder cells and/or ECM [5]. Additionally, the undefined nature of serum and other xenogeneic components limits the ability to tune the cellular microenvironment and in turn affects the efficiency and reproducibility of the protocol [16, 19]. Furthermore, these xenogeneic components limit the clinical translation potential owing to potential risk of transmission of animal pathogens, and ectopic expression of immunogenic minor histocompatibility antigens that could lead to xenogeneic rejection [16]. Hence, large-scale production of ECs from hESCs for various research and clinical applications would require: (1) efficient induction of hESCs towards endothelial lineage and specifically towards different arterial and venous ECs, and (2) elimination or reduction of xenogeneic products.

A reliable approach to generate ECs from hESCs would be to recapitulate the embryonic vasculogenesis under defined conditions based on a thorough understanding of the key developmental events and signaling pathways controlling them. In this study, we established a stepwise differentiation of hESCs to arterial and venous ECs through stages reminiscent of Brachyury^+^ primitive streak (PS), VEGFR2^+^ early mesoderm/lateral plate mesoderm, CD34^+^CD31^+^ (VEGFR2^+^) endothelial progenitors and then to arterial (CD31^+^/NRP1^+^/DLL4^+^) and venous (CD31^+^/NRP2^+^/EphB4^+^) ECs under feeder- and serum-free conditions. These endothelial phenotypes displayed differences in transcriptome and secretome profiles, they were nontumorigenic and formed functional blood vessels that integrated with the host circulation and maintained their respective phenotypes in vivo.

## Methods

### Culture of hESCs under feeder-free and serum-free conditions

For feeder- and serum-free culture, H1- and H9-hESCs (WiCell Research Institute, Madison, WI, USA) were cultured in chemically defined medium (mTeSR™1; StemCell Technologies) on Matrigel-coated plates (356230, BD Biosciences) as previously described [20]. Briefly, 70–80 % confluent hESCs were passaged after treatment with 1 mg/ml dispase (Invitrogen) for 5 minutes at 37 °C. The hESC colonies were dispersed into small clumps and re-plated onto Matrigel-coated plates. The pluripotency status of the hESCs was confirmed by immunocytochemical expression of OCT4, SSEA3/4, TRA-1-60, TRA-1-81 and alkaline phosphatase (Additional file 1: Figure S1).

### Directed differentiation of hESCs under chemically defined conditions

**Fig. 1:**
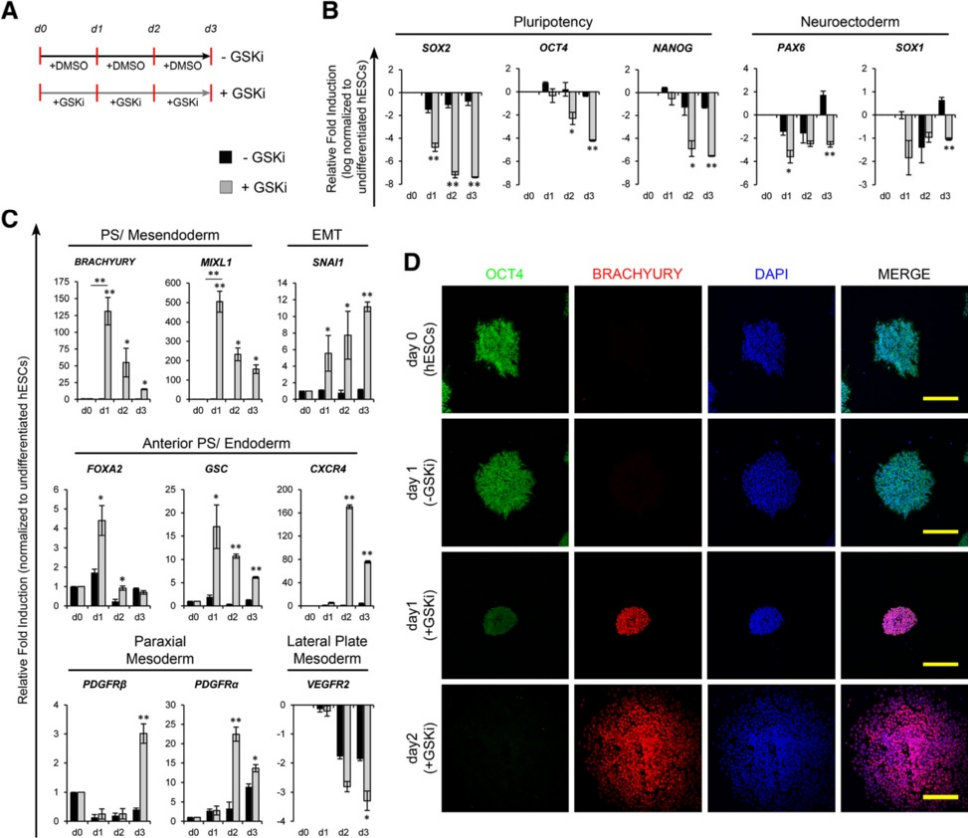
Time course analysis of expression of genes associated with pluripotency and early differentiation of human embryonic stem cells (*hESCs*) in response to inhibition of glycogen synthase kinase-3 (*GSK-3*). **a** Schematic representation of differentiation of hESCs with or without the inhibition of GSK-3 using CHIR99021 (*±GSKi*). Real-time RT-PCR analysis of markers associated with pluripotency and neuroectoderm (**b**), primitive streak (*PS*)/mesendoderm, epithelial–mesenchymal transition (*EMT*), anterior PS/endoderm, and mesodermal subsets (**c**) after differentiation of hESCs with (*grey bars*) or without (*black bars*) GSKi. For all gene expression plots, expression levels were normalized to corresponding *β-ACTIN* values and are shown relative to undifferentiated hESCs. The expression levels of *SOX2, OCT4, NANOG, PAX6, SOX1* and *VEGFR2* were log-normalized to reveal the amount of downregulation in relation to undifferentiated hESCs. Error bars show standard deviations; n ≥ 3. *p < 0.05, **p < 0.01, versus without GSKi. **d** Confocal immunofluorescence micrographs show expression of OCT4 and BRACHYURY after differentiation with or without GSKi. Scale bars = 200 μm. *DMSO* Dimethyl sulfoxide,

**Fig. 2:**
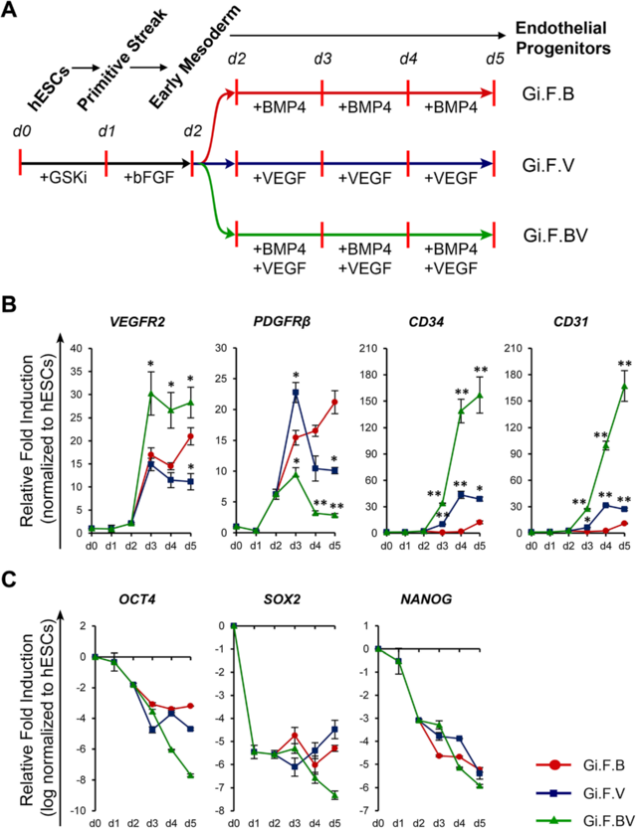
Kinetics of transcripts associated with early endothelial induction and pluripotency of human embryonic stem cells (*hESCs*). **a** Schematic representation of differentiation of H1-hESCs towards endothelial progenitors by sequential treatment with CHIR99021 (*+GSKi*) and basic fibroblast growth factor (*bFGF*) for 24 hours each followed by exposure to bone morphogenetic protein 4 (*BMP4*) and/or vascular endothelial growth factor (*VEGF*). **b** Kinetics of expression of *VEGFR2*, *CD34* and *CD31* upon induction with BMP4 (*Gi.F.B*), VEGF (*Gi.F.V*) and BMP4 + VEGF (*Gi.F.BV*) over a differentiation period of 5 days. **c** Time course expression kinetics of pluripotency genes (*OCT4*, *SOX2*, *NANOG*) over 5 days of differentiation among the three differentiation conditions. For gene expression plots in (**b**) expression levels were normalized to corresponding *β-ACTIN* values and are shown relative to undifferentiated hESCs, while for those in (**c**) the expression levels were log-normalized to reveal the amount of downregulation in relation to undifferentiated hESCs. Error bars show standard deviations; n ≥ 3. *p < 0.05, **p < 0.01, versus Gi.F.B

After incubation with dispase (1 mg/ml; 5 minutes), undifferentiated hESC colonies of size ~300–500 cells per colony were gently cut into small squares using a 200 μl gel loading pipette tip under a stereomicroscope (Olympus). These cut hESC colonies were gently aspirated and seeded onto 4 μg/cm^2^ human plasma fibronectin (GIBCO)-coated plates (seeding density: ~2 hESC colonies/cm^2^). The pluripotency status of hESCs seeded onto fibronectin-coated plates for 24 hours was investigated by real-time reverse-transcriptase polymerase chain reaction (RT-PCR) and immunocytochemical staining (Additional file 1: Figure S2). These hESC colonies on fibronectin were maintained in mTeSR™1 for 24 hours, after which the cells were gently washed with DMEM:F12 (Invitrogen) and differentiated in chemically defined, serum-free, animal component-free medium [21] (STEMdiff™APEL™; StemCell Technologies) supplemented with appropriate factors as depicted in Figs. 1 and 2. To induce hESCs towards PS, the hESCs were exposed to glycogen synthase kinase-3 (GSK-3) inhibitor (CHIR99021; 5 μM; Stemgent) for 24 hours as previously reported [17, 22]. After 24-hour exposure of hESCs to CHIR99021, mesodermal induction of PS was carried out using basic fibroblast growth factor (bFGF) (50 ng/ml) with or without FGF receptor inhibitor (PD173074; 0.1 μM), and endodermal differentiation of PS was induced using Activin-A (50 ng/ml) with or without Activin and transforming growth factor (TGF)-β signaling inhibitor SB431542 (10 μM) as illustrated in Additional file 1 (Figures S3 and S4). Following induction of PS, mesodermal and endothelial induction was carried out in the presence of bFGF (50 ng/ml; R&D systems) for 24 hours followed by 72 hours of bone morphogenetic protein 4 (BMP4; 25 ng/ml; R&D systems) and VEGF (50 ng/ml; GIBCO) (Fig. 2a).

### Differentiation of endothelial progenitors (CD34^+^CD31^+^ cells) to endothelial subtypes in feeder-free, serum-free conditions

After 5 days of differentiation in STEMdiff™APEL™, CD34^+^CD31^+^ cells were isolated by flow cytometry assisted sorting (FACS), and plated onto 2 μg/cm^2^ human plasma fibronectin-coated plates (12,000 cells per cm^2^) and cultured in endothelial serum-free medium (ESFM; GIBCO) with media changes every 2–3 days. Venous differentiation was induced using 10 ng/ml epidermal growth factor (EGF; R&D Systems), and 20 ng/ml bFGF. For arterial differentiation, the medium was supplemented with 10 ng/ml EGF, 20 ng/ml bFGF, and 10 ng/ml VEGF. The endothelial cells were passaged using accutase (Invitrogen) when 70–80 % confluent and characterized after 3 to 5 passages.

### In vivo studies

All experiments involving handling and manipulation of animals were approved by the Institutional Animal Care and Use Committee, National University of Singapore, and the Institutional Review Board, National University of Singapore. To assess the angiogenic potential of hESC-derived arterial and venous ECs, 1 × 10^6^ hESC arterial ECs (Art-ECs) or hESC venous ECs (Ven-ECs) were suspended in 200 μl of growth factor-reduced Matrigel and injected subcutaneously in each side of the dorsal region of 8- to 9-week-old nonobese diabetic (NOD)/severe combined immunodeficiency (SCID) mice [NOD.CB17-*Prkdc*^*scid*^/J] (two implants per mouse; three mice per experimental condition). Matrigel plug without cells served as control. After 2 weeks, the Matrigel plug was harvested with the surrounding tissues and processed for histological analysis.

To assess the safety of hESC-derived ECs, 1 × 10^6^ hESCs, hESC-Art-ECs, or hESC-Ven-ECs were suspended in 200 μl of phosphate-buffered saline and injected subcutaneously in each side of the dorsal region of 6- to 8-week-old NOD/SCID mice [NOD.CB17-*Prkdc*^*scid*^/J] (two implants per mouse; three mice per experimental condition). After 4 months or formation of tumor mass ≥1.5 cm in size (whichever was earlier), the mice were sacrificed and the tissue around the point of injection was harvested and processed for histological analysis.

### Supplementary methods

For additional methods refer to Additional file 1. Information related to list of primers and antibodies used in the study are presented in Additional file 1.

## Results

For all the differentiation studies the H1-hESC line was used primarily, and the robustness of the protocol verified using H9-hESCs.

### Temporal emergence of primitive streak capable of commitment to mesoderm and endoderm

In early embryogenesis, mesoderm that gives rise to cells of the vascular lineage arises through an epithelial–mesenchymal transition (EMT) of epiblast cells in the region of PS. For differentiation to PS, we modified our previously published protocol [17], using plasma fibronectin as substrate and CHIR99021 (5 μM) to inhibit GSK-3 under chemically defined conditions. During the induction of PS, we observed a marked downregulation of the pluripotency gene *SOX2* within 24 hours, followed by *OCT4* and *NANOG*, and the neuroectoderm-associated genes (*PAX6, SOX1*) (Fig. 1a and b). In contrast to the downregulation of neuroectodermal genes, the PS-related genes (*BRACHYURY*, *MIXL1*) were upregulated synchronously along with anterior PS genes (*FOXA2, GSC*) and peaked at day 1 of differentiation (Fig. 1c). Furthermore, the temporal expression of PS-related genes was accompanied by upregulation of the gene involved in EMT (*SNAI1*). The expression of BRACHYURY along with OCT4 after 24 hours of inhibition of GSK-3 was confirmed by immunofluorescence (Fig. 1d). Temporal expression of Brachyury accompanied with EMT suggests the emergence of Brachyury^+^ PS-like cells at early stages of inhibition of GSK-3 as previously reported [17, 23–25].

Cells of the PS have the ability to commit to mesoderm and endodermal progenies depending on the balance between bFGF, Activin and BMP4 signaling [3, 17, 23, 26]. We next sought to ascertain the differentiation capacity of PS towards mesoderm and endoderm, as illustrated in Additional file 1 (Figures S3 and S4). Real-time PCR analysis revealed that induction with bFGF resulted in modest upregulation of lateral and paraxial mesodermal transcripts (*VEGFR2, PDGFRα, PDGFRβ*) accompanied by downregulation of PS and endoderm-related transcripts (*FOXA2*, *GSC*, *CXCR4*) (Additional file 1: Figure S3). On the contrary, treatment of PS with Activin-A resulted in marked upregulation of endodermal transcripts and downregulation of PS and mesoderm-related transcripts, while these findings were abrogated in the presence of SB431542 (Additional file 1: Figure S4). Collectively, these observations demonstrate the potential of hESCs differentiated to PS under the influence of GSK-3 inhibition (for 24 hours) to commit towards mesoderm or endoderm depending on the culture milieu provided.

### VEGF is essential and sufficient for endothelial commitment from early mesodermal cells, and BMP4 is synergistic

**Fig. 3:**
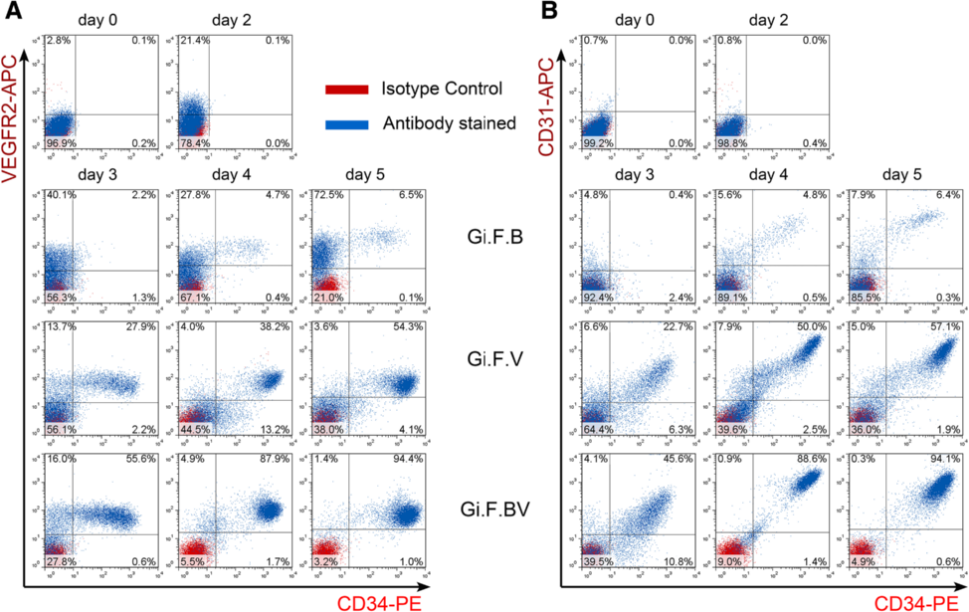
Flow cytometry analysis of induction of H1-hESCs towards endothelial lineage. Representative flow cytometry overlays display the kinetics of co-expression of vascular endothelial growth factor receptor-2 (*VEGFR2*) and CD34 (**a**), and CD31 and CD34 (**b**) upon induction of H1-hESCs with BMP4 (*Gi.F.B*), VEGF (*Gi.F.V*) and BMP4 + VEGF (*Gi.F.BV*) over a differentiation period of 5 days

Inhibition of GSK-3 followed by bFGF exposure drives the hESCs towards lateral plate mesoderm as evidenced by the upregulation of *VEGFR2* and downregulation of PS and endoderm-related genes. We next sought to investigate the potential of these lateral plate mesoderm cells to commit towards endothelial lineage. Differentiation was performed using BMP4 and VEGF after initial treatment with CHIR99021 and bFGF as illustrated in Fig. 2a. The kinetics of differentiation towards endothelial lineage was monitored using VEGFR2 (an early marker for lateral plate mesoderm-derived progenitors), CD34 (early marker for progenitors with potential to differentiate towards hemato-endothelial lineage), CD31 (pan-endothelial lineage marker) and platelet-derived growth factor (PDGF)Rβ (early marker for paraxial mesoderm). Real-time PCR analysis (Fig. 2b) revealed BMP4 supplementation resulted in upregulation of *VEGFR2*, but had minimal effect on the expression levels of *CD34* and *CD31*. On the contrary, treatment with VEGF resulted in a modest increase in the transcript levels of *CD34*, *CD31* and *VEGFR2* and downregulation of *PDGFRβ*, while combined modulation with BMP4 and VEGF resulted in marked upregulation of *CD34*, *CD31* and *VEGFR2* and marked downregulation of *PDGFRβ*. Furthermore, the pluripotency markers were markedly downregulated under all the three differentiation conditions (Fig. 2c). In accordance with the real-time PCR data, time-course flow cytometry analysis (Fig. 3) revealed the gradual emergence of a VEGFR2^+^ population with BMP4 supplementation, but only a small subset of this population co-express CD34 (~6.5 % on day 5), while the addition of VEGF resulted in the gradual appearance of CD34^+^ cells that co-express VEGFR2 and CD31 and account for ~54 % of the differentiated cells by the fifth day of differentiation. Furthermore, combined treatment with BMP4 and VEGF resulted in a robust emergence of CD34^+^VEGFR2^+^/ CD34^+^CD31^+^ cells which accounted for ~95 % of the differentiated cells by the fifth day of differentiation. In all three differentiation conditions, time-course flow cytometry plots also reveal the temporal emergence of a VEGFR2^+^ population that gradually attains CD34 and CD31 positivity. These findings suggest the role of VEGF in induction to lateral plate mesodermal progenitors (VEGFR2^+^ cells) and further to endothelial progenitors (CD34^+^CD31^+^VEGFR2^+^ cells) and a synergistic role of BMP4 resulting in robust commitment to the endothelial lineage. We verified the robustness of the protocol using H9-hESCs which also yielded ~90 % of cells positive for VEGFR2, CD34 and CD31 (Additional file 1: Figure S5). In conclusion, step-wise treatment of hESCs with GSK-3 inhibitor, bFGF followed by BMP4 and VEGF results in sequential emergence of Brachyury^+^ PS , VEGFR2^+^ early mesoderm/lateral mesoderm cells followed by robust commitment to endothelial lineage yielding ~90–95 % of CD34^+^CD31^+^(VEGFR2^+^) endothelial progenitor cells within a differentiation span of 5 days under feeder-free, and chemically defined conditions.

### Terminal differentiation of CD34^+^CD31^+^ endothelial progenitors to arterial and venous endothelial cells under serum-free conditions

**Fig. 4:**
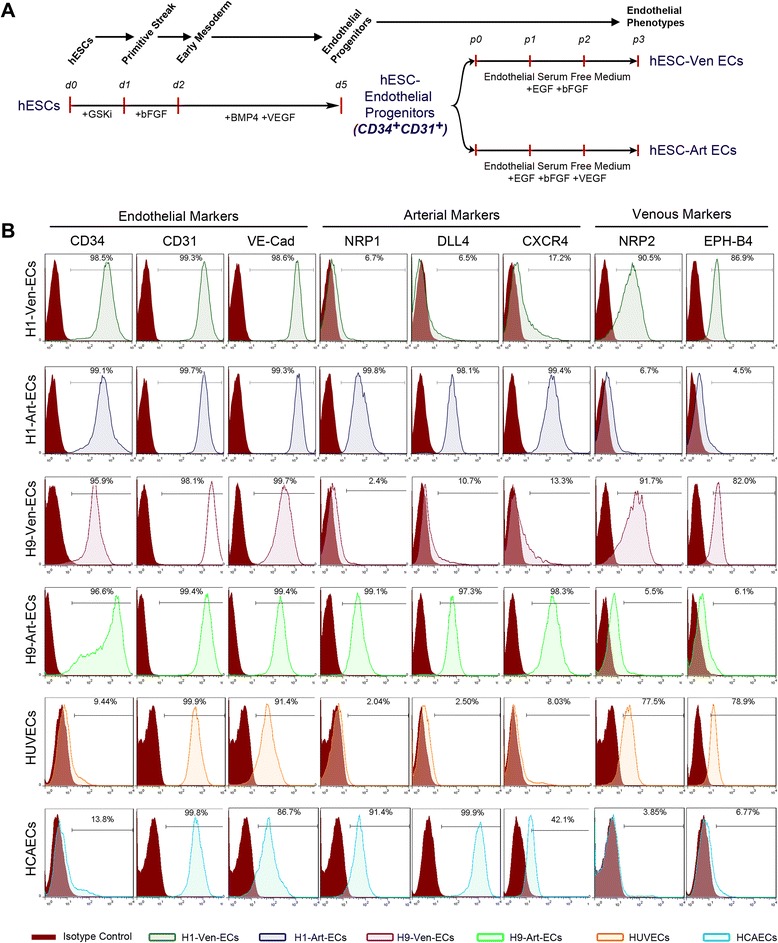
Flow cytometry characterization of terminal differentiation of CD34^+^CD31^+^ endothelial progenitors to venous and arterial endothelial cells under serum-free conditions. **a** Schematic representation of differentiation of hESCs to endothelial progenitors and further differentiation towards venous ECs (*hESC-Ven-ECs*) and arterial ECs (*hESC-Art-ECs*). **b** Representative flow cytometry histogram overlays represent the expression of pan-endothelial, arterial and venous markers among H1-Ven-ECs, H1-Art-ECs, H9-Ven-ECs, H9-Art-ECs, HUVECs, and HCAECs. *bFGF* Basic fibroblast growth factor, *BMP4* Bone morphogenetic protein 4, *EGF* Epidermal growth factor, *GSKi* Glycogen synthase kinase inhibitor (CHIR99021), *HCAEC* Human coronary artery endothelial cells, *hESC* Human embryonic stem cells, *HUVEC* Human umbilical vein endothelial cells, *VEGF* Vascular endothelial growth factor

**Fig. 5:**
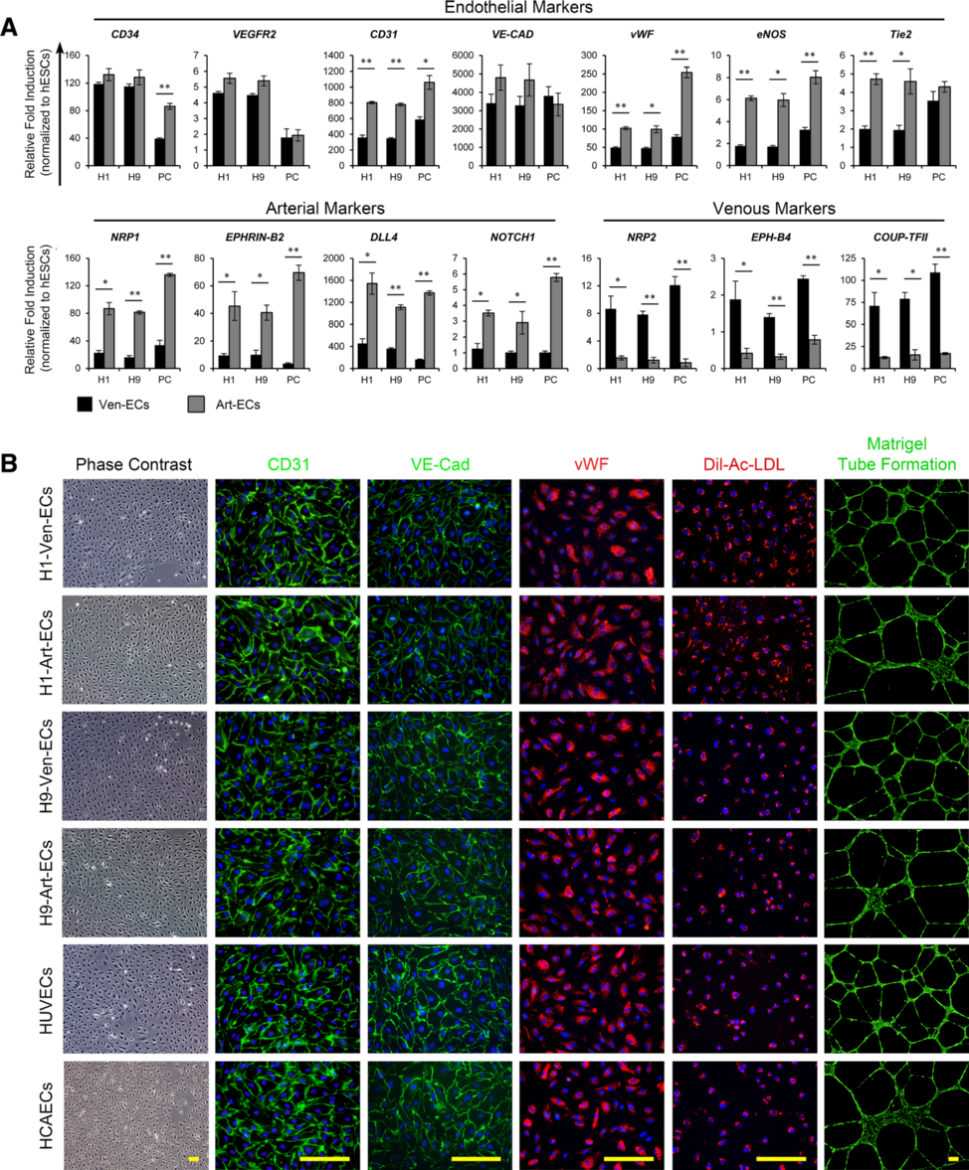
Characterization of hESC-derived venous and arterial endothelial cells. **a** Profiles of transcripts related to endothelial, arterial and venous phenotypes among Ven-ECs and Art-ECs derived from H1-hESCs, H9-hESCs and primary cells (*PC*: HUVECs and HCAECs). Gene expression levels were normalized to corresponding *β-ACTIN* values and are represented as relative to undifferentiated hESCs. **b** Representative photomicrographs of H1-Ven-ECs, H1-Art-ECs, H9-Ven-ECs, H9-Art-ECs, HUVECs, and HCAECs show the cobblestone morphology of ECs under phase contrast microscopy, and immunofluorescence images demonstrate the expression of pan-endothelial markers CD31, VE-Cadherin, and von Willebrand factor (*vWF*), uptake of Dil-acetylated low-density lipoprotein (*Dil-Ac-LDL*) and formation of cord-like structures over Matrigel (Green-CalceinAM). Scale bars = 150 μm. Error bars show standard deviation; n ≥ 3. *p < 0.05, **p < 0.01. *Art-EC* Arterial endothelial cells, *HCAEC* Human coronary artery endothelial cells, *hESC* Human embryonic stem cells, *HUVEC* Human umbilical vein endothelial cells, *Ven-EC* Venous endothelial cells

Under serum-containing conditions, high concentrations (50 ng/ml) of VEGF have been reported to aid arterial differentiation, while lower concentrations (10 ng/ml) aid venous commitment of mouse ESCs and human iPSCs [27–29]. However, differentiation of hESCs to arterial and venous ECs and, specifically, differentiation under serum-free conditions has not been reported so far. The CD34^+^CD31^+^ endothelial progenitors were sorted and further differentiated towards endothelial subtypes in serum-free conditions using commercially available ESFM. Serum-containing endothelial medium typically requires supplementation with FBS (2–5 %), insulin, heparin, ascorbic acid, hydrocortisone, insulin-like growth factor, bFGF, EGF and VEGF, but the serum-free endothelial medium as per manufacturer’s instructions requires supplementation with bFGF (20 ng/ml) and EGF (10 ng/ml) only. Hence we initially carried out the differentiation of the CD34^+^CD31^+^ cells in ESFM supplemented with bFGF and EGF for 3–6 passages (Fig. 4a). Differentiation under these conditions yielded 98–99 % CD34^+^/CD31^+^/VE-CAD^+^ ECs (Fig. 4b). Real time RT-PCR analysis demonstrated upregulation of all transcripts associated with endothelial lineage (Fig. 5a). Additionally, immunocytochemistry revealed the expression of *CD31*, *VE-CAD* and *vWF* (Fig. 5b). Further analysis into the arterial and venous phenotype markers showed almost 80–90 % of the cells to be positive for venous markers (NRP2, EPH-B4) while only ~2–10 % of the cells differentiated from H1/H9-hESCs expressed NRP1 and DLL4, and ~13–17 % expressed CXCR4 (Fig. 4b). The endothelial, arterial and venous marker expression profiles were similar to those expressed by human umbilical vein endothelial cells (HUVECs) (Figs. 4 and 5).These observations suggest the commitment of CD34^+^CD31^+^ cells towards venous endothelial phenotype and these would be referred to as hESC-Ven-ECs (H1/H9).

VEGF has been reported as critical for vascular patterning governing the specification towards arterial phenotype through a cascade of signaling events involving Shh, Notch, Dll4 and Ephrin-B2 [3, 30–32]. Similar studies on mouse ESCs [29, 30], human adult progenitors [33] and human iPSCs [28] have shown high concentrations of VEGF favoring arterial specification. To ascertain if the CD34^+^CD31^+^ cells had the potential to commit towards arterial phenotype, we additionally supplemented the ESFM with VEGF in addition to EGF and bFGF. Surprisingly, when the CD34^+^CD31^+^ cells were exposed to high concentrations of VEGF (50 ng/ml), we observed apoptosis of the cells. Titration of VEGF concentration and analysis of apoptotic cell death using AnnexinV-Propidium iodide staining revealed the occurrence of cellular apoptosis in a dose-dependent manner with the optimal concentration around 5–10 ng/ml VEGF (Additional file 1: Figure S6). Differentiation of H1/H9-hESC-derived CD34^+^CD31^+^ cells in the presence of 10 ng/ml VEGF for 3–6 passages, yielded almost 95–99 % of CD34^+^/CD31^+^/VE-CAD^+^ ECs (Fig. 4b). Flow cytometry analysis showed that these ECs were also positive for arterial markers (NRP1, DLL4, CXCR4) and only 5–6 % of the cells were positive for venous markers (NRP2, EPH-B4) (Fig. 4b). When compared to Ven-ECs, H1/H9-hESC-derived endothelial progenitors differentiated under the influence of VEGF displayed significantly higher levels of *CD31*, *vWF*, eNOS and *Tie2* (Fig. 5a). Real-time PCR analysis also revealed that these cells had significantly higher levels of arterial markers and lower levels of venous endothelial markers (Fig. 5a). The endothelial, arterial and venous marker expression profiles were similar to those expressed by human coronary artery endothelial cells (HCAECs) (Figs. 4 and 5). The expression profile of the cells differentiated under the influence of low concentrations of VEGF indicate the commitment of CD34^+^CD31^+^ cells towards arterial endothelial phenotype and these would be referred to as hESC-Art-ECs (H1/H9). Furthermore, both hESC-Art-ECs and hESC-Ven-ECs were functional in terms of their ability for acetylated low-density lipoprotein uptake and formation of cord-like structures over Matrigel (Fig. 5b).

Studies have shown that ECs commit to arterial or venous fate in early embryonic development. However, studies suggest that ECs display a certain degree of plasticity and express characteristics of alternative endothelial phenotypes [34–36]. This degree of plasticity of endothelial phenotypes is poorly understood, though studies suggest the role of hemodynamic forces, microenvironment, smooth muscle coverage, wounding and pathological conditions [34–38]. Hence, to examine whether these hESC-derived ECs are committed to an arterial or venous fate, we cross-cultured the hESC-derived arterial ECs in venous media conditions (i.e., in the absence of VEGF) and hESC-derived venous ECs in arterial media conditions by supplementing the medium with VEGF. Both H1- and H9-hESC-derived arterial ECs, upon culture under venous conditions for two passages, resulted in a 15–25 % reduction in the number of ECs expressing arterial markers (NRP1 and DLL4) and were associated with a 12–20 % increase in the number of ECs expressing venous markers (NRP2 and EPH-B4) (Additional file 1: Figure S7). Similarly, culture of H1- and H9-hESC-derived venous ECs under arterial conditions for two passages resulted in a 15–20 % reduction in the number of ECs expressing venous markers and was associated with a 7–15 % increase in the number of ECs expressing arterial markers. Hence, the changes in culture conditions resulted in transition of a small subset of the arterial ECs to venous phenotype and vice versa.

In summary, we developed a protocol that results in rapid and efficient differentiation of hESCs to CD34^+^CD31^+^ endothelial progenitors with the potential to commit to arterial and venous endothelial phenotypes under feeder- and serum-free conditions through modulating the concentration of VEGF.

### Intrinsic differences in cell migration and angiocrine secretome profiles of hESC-derived arterial and venous ECs

**Fig. 6:**
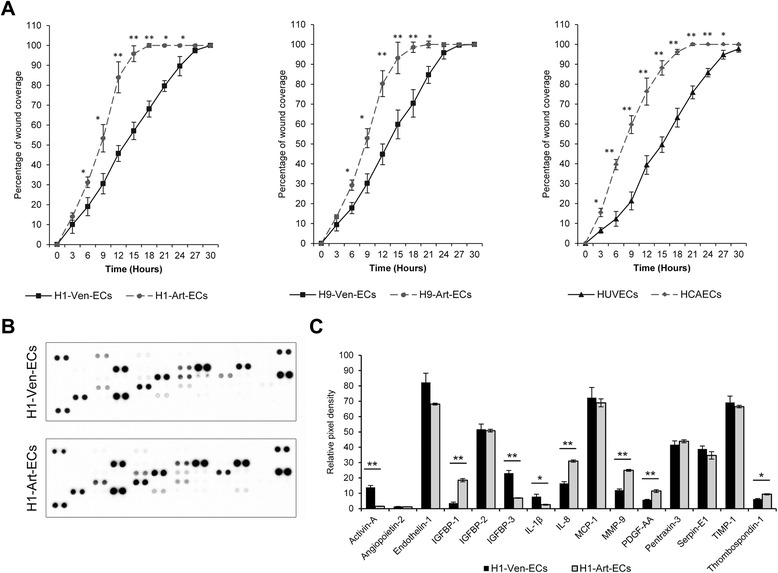
Assessment of cell migration and survey of angiocrine secretome profiles of hESC-derived arterial and venous ECs. **a** Graphical representation of kinetics of wound coverage among H1-Ven-ECs and H1-Art-ECs (*left panel*), H9-Ven-ECs and H9-Art-ECs (*middle panel*), HUVECs, and HCAECs (*right panel*). Error bars show standard deviation; n = 3. *p < 0.05, **p < 0.01. **b** Representative scans of angiogenesis antibody arrays demonstrating the secretion of various angiocrine factors by H1-Ven-ECs and H1-Art-ECs. Array images are obtained from 10-minute exposure of X-ray film. (Refer to Additional file 1 for co-ordinates of the antibody array). **c** Graphical representation of the relative amounts of selected angiocrine factors that show significant differences among the H1-hESC derived arterial and venous ECs. The bars represent relative amounts of factors secreted based on densitometric analysis of relative pixel density of the blots. Error bars show the standard deviation of two independent experiments. *p < 0.05, **p < 0.01. *Art-EC* Arterial endothelial cells, *HCAEC* Human coronary artery endothelial cells, *hESC* Human embryonic stem cells, *HUVEC* Human umbilical vein endothelial cells, *IGF* Insulin-like growth factor, *IL* Interleukin, *MCP* monocyte chemoattractant protein, *MMP* Matrix metalloproteinase, *PDGF* Platelet-derived growth factor, *TIMP* Tissue inhibitor of matrix metalloproteinase, *Ven-EC* Venous endothelial cells

ECs interact with the local microenvironment and support tissue regeneration after injury through revascularization of the newly healed tissue and expression of various trophic growth factors, known as angiocrine factors, which lead to proliferation and migration of ECs from pre-existing blood vessels. To assess cell migration, in vitro wound closing assays were performed over a period of 24 hours. Compared to H1-Ven-ECs, the H1-Art-ECs displayed faster closure of the wound area resulting in a complete wound closure by H1-Art-ECs in ~15 hours in contrast to ~27 hours taken by H1-Ven-ECs (Fig. 6a). Similarly, the H9-Art-ECs and HCAECs displayed faster closure of the scratch wound compared to respective venous phenotypes (Fig. 6a). ECs pertaining to distinct vascular beds have recently been reported to display significant differences in expression profiles of various transcriptomes, angiocrine factors and surface markers [39]. However, the distinct expression of various angiocrine factors by different endothelial phenotypes of hESC origin still needs to be explored. To probe into the profile of various angiocrine factors secreted by hESC-derived ECs, we used angiogenesis antibody array of 55 different proteins related to angiogenesis, growth factors and related proteins, cytokines, proteases and their inhibitors, neurotrophic factors, and anti-angiogenic factors. Both H1-Art-ECs and H1-Ven-ECs displayed marked induction of endothelin-1, insulin-like growth factor-binding protein-2 (IGFBP-2), monocyte chemoattractant protein-1 (MCP-1), pentraxin-3, serpin-E1 and tissue inhibitor of matrix metalloproteinase-1 (TIMP-1) (Fig. 6; Additional file 1: Figure S7 and Table S1). However, the conditioned media of H1-Ven-ECs had significantly higher levels of Activin-A, IGFBP-3, and interleukin-1β compared to H1-Art-ECs (Fig. 6c). On the contrary, the media conditioned by H1-Art-ECs had significantly higher levels of IGFBP-1, interleukin-8, matrix metalloproteinase-9 (MMP-9), PDGF-AA and thrombospondin-1 (TSP-1) compared to that of H1-Ven-ECs (Fig. 6c). These results emphasize the existence of intrinsic differences between arterial and venous endothelial phenotypes. However, further studies on the differences in secretome profiles among the two endothelial phenotypes and their in vivo relevance are needed to understand the role of endothelial heterogeneity.

### Transplantation of hESC-derived arterial and venous ECs result in formation of microvessels

**Fig. 7:**
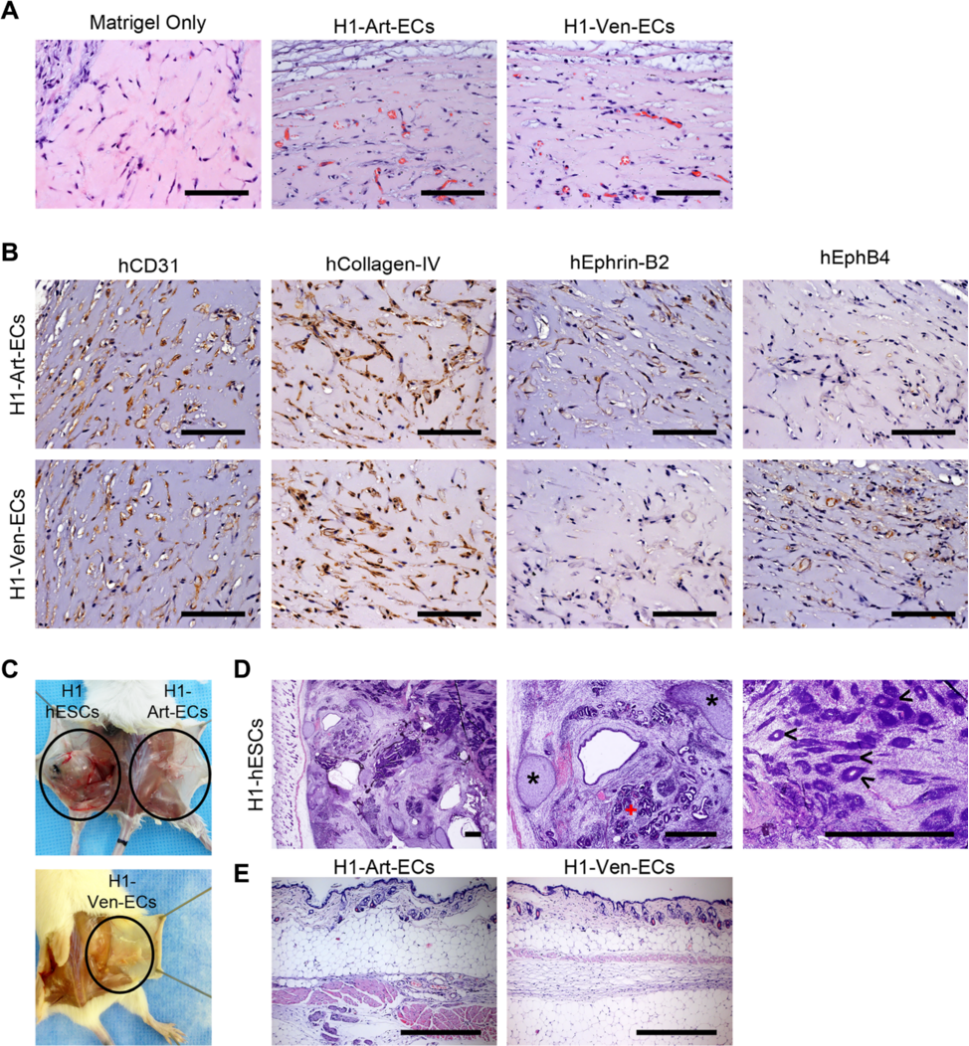
In vivo safety and functionality assessment of hESC-derived arterial and venous endothelial cells. **a** Representative hematoxylin and eosin (H&E) stained micrographs of Matrigel plug study showing absence of microvessels in Matrigel-only control, while the others show the formation of perfused microvessels by engrafted hESC-derived arterial and venous ECs. **b** Histochemical micrographs of microvessels formed by hESC-derived arterial and venous ECs stained for hCD31, hCollagen-IV, hEphrin-B2 and hEph-B4. **c** Representative photographs showing the presence or absence of teratoma formation in immunodeficient mice. **d** Representative H&E stained micrographs showing the formation of teratoma by H1-hESCs with evidence of differentiation to derivatives of three germ layers: ectoderm (hair follicles, <), mesoderm (cartilage, *), and endoderm (glandular structures, +). **e** Representative H&E stained micrographs showing the absence of teratoma formation by H1-hESCs derived arterial and venous ECs. Scale bars in (**a**) and (**b**) = 100 μm; in (**d**) and (**e**) = 500 μm. *Art-EC* Arterial endothelial cells, *hESC* Human embryonic stem cells, *Ven-EC* Venous endothelial cells

After assessment of the in vitro functionality of the hESC-derived arterial and venous ECs, we investigated whether these ECs have the ability to form functional microvessels upon transplantation, by injecting ECs suspended in Matrigel subcutaneously into the dorsal region of immunodeficient mice. Matrigel plugs implanted without cells exhibited no evidence of microvessels within the matrix (Fig. 7a). On the other hand, the Matrigel plugs implanted along with hESC-Art-ECs or hESC-Ven-ECs showed the presence of microvessels within the matrix. Additionally, these microvessels were seen to be connected to the host circulation, as evidenced by the presence of red blood cells within the lumen (Fig. 7a). Furthermore, these microvessels were reactive for anti-human CD31 and anti-human collagen-IV, indicating that they were of human origin (Fig. 7b). Furthermore, the hESC-Art-ECs and hESC-Ven-ECs largely maintained their respective phenotypes in vivo, as evidenced by the expression of Ephrin-B2 and Eph-B4, respectively (Fig. 7b). However, few microvessels formed by hESC-Art-ECs showed the expression of venous markers and vice versa.

To determine if the hESC-derived arterial and venous ECs have tumorogenic potential, we transplanted H1-hESC-derived arterial and venous ECs and their cell source (H1-hESCs) into immunocompromised mice. All the mice injected with H1-hESCs developed teratomas within 3–4 months of transplantation, while none of those that received either of the ECs showed sign of teratoma for the same period of time (Fig. 7c-e). Hence, the hESC-derived arterial and venous ECs differentiated under feeder- and serum-free conditions were functional and non-tumorogenic *in-vivo*.

## Discussion

In the present study, we demonstrate a novel and efficient protocol for monolayer differentiation of hESCs towards arterial and venous ECs under feeder- and serum-free conditions. These hESC-derived arterial and venous ECs demonstrate differences in gene expression and angiocrine secretion profiles, and form functional microvessels in vivo. Despite tremendous progress in the differentiation of hESCs to ECs, current protocols are inefficient predominantly due to a poorly controlled differentiation microenvironment and lack of the concept of a lineage-directed approach [5]. We [17] and others [23, 24, 40–43] have demonstrated that activation of the Wnt/β-Catenin pathway with or without BMP4 and Activin-A efficiently drives the differentiation of PSCs towards mesoderm and endoderm lineages. Cao et al*.* demonstrated the robust differentiation of PSCs to cardiovascular progenitors upon combined treatment with BMP4, CHIR99021 and ascorbic acid in a chemically defined medium under feeder- and serum-free culture conditions [44]. Similarly, a recent report demonstrates a robust commitment of human iPSCs towards mesoderm in the presence of the GSK-3 inhibitor CHIR99021 followed by induction to cardiac progenitors upon β-Catenin siRNA knockdown, suggesting a dual function of the Wnt/β-Catenin pathway during early cardiovascular development [45]. In this study, we directed the hESCs towards lateral plate mesoderm under feeder-free and chemically defined conditions through short-term inhibition of GSK-3 followed by treatment with bFGF and BMP4. Currently available protocols to differentiate hESC and hiPSCs towards endothelial lineage generally requires 10–15 days of differentiation to achieve a modest 2–40 % of progenitor cells committed to endothelial lineage [5]. Recent reports show commitment of hESCs to endothelial lineage with ~50 % efficiency by using GSK-3 inhibition and treatment with BMP4 at early stages of differentiation, followed by treatment with BMP4 and inhibition of Notch/TGFβ signaling pathways [17, 40]. Though a detailed comparison with published protocols was not undertaken, our current findings suggest a robust commitment (90–95 %) towards endothelial lineage within a differentiation span of 5 days. Furthermore, the differentiation protocol primarily results in generation of CD34^+^CD31^+^ cells, in comparison to previously published methods which generates a mixture of CD34^+^CD31^−^, CD34^−^CD31^+^ CD34^+^CD31^+^ and CD34^−^CD31^−^ cells [46, 47]. The robustness of our endothelial differentiation protocol was verified among two different hESC lines; however, it needs further validation using other hESC lines and iPSCs.

Distinction of arterial and venous endothelial phenotypes seems to occur quite early in the development even before the onset of circulation, wherein VEGF, Shh, and Notch signaling have been suggested to play a crucial role [31, 32]. In vitro studies using mouse PSCs [29, 48] indicate the expression of arterial markers such as *Dll4, EphrinB2* and *Notch4* in response to high concentrations of VEGF (50 ng/ml), while lower concentrations (10 ng/ml) resulted in upregulation of the venous marker *COUP-TFII*. Additionally, the VEGF-mediated arterialization was reported to be further enhanced by the addition of adrenomedullin [27, 48] and these effects were blocked in the absence of Notch signaling [27, 29]. These findings from in vitro models correlate with the findings of in vivo animal models highlighting the coordinated activation of VEGF-Notch signaling in arterial specification. Though tremendous amounts of information have been obtained from in vitro human models, the heterogeneity of ECs and/or derivation of tissue-specific ECs have been investigated by very few studies. Human cord blood-derived endothelial progenitors [49] and hPSCs [50, 51] upon co-culture with neural cells differentiate to brain-specific ECs and express markers and functions related to the blood–brain barrier. Recent studies based on plastic conversion of fibroblasts to ECs and iPSC-derived ECs reported the pool of terminally differentiated ECs as being heterogeneous in terms of expression of arterial, venous and lymphatic markers [52, 53]. However, distinct specification towards either phenotype is not investigated. Only two studies using human adult progenitors [33] and human iPSCs [28] have elucidated the arterial–venous specification of human progenitor/stem cells to date. Aranguren et al*.* reported that a high concentration of VEGF (100 ng/ml) induced arterial differentiation of human bone marrow-derived multipotent adult progenitor cells [33]. Furthermore, the arterial induction was reported to be enhanced by supplementation with Dll4 and Shh, while blockade of Notch and/or Shh led to attenuation of arterial differentiation and upregulation of venous markers. Similarly, Rufaihah et al*.*, using human iPSCs, demonstrated arterial differentiation by high VEGF concentration coupled with cAMP, and induction of venous specification under low concentrations of VEGF [28]. In summary, all the above studies in mouse and human in vitro models indicate the dose-dependent effect of VEGF in arterial–venous specification of ECs; however, these studies were performed under serum-containing conditions.

Inclusion of xenogeneic products (like Matrigel, FBS, murine stromal cells) in the culture milieu could influence the differentiation outcome and limit the ability to tune the microenvironment due to the presence of unknown/poorly defined factors [16]. To eliminate or reduce the use of xenogeneic products, we have developed a novel feeder- and serum-free protocol for differentiation of hESCs to endothelial subtypes. Under serum-free conditions, we found higher concentrations of VEGF (25–100 ng/ml) caused apoptosis of the cells, while at lower concentrations (10 ng/ml) it directs towards the arterial phenotype and in its absence favors the venous fate. Spatially different response of hESC-derived endothelial progenitors to various concentrations of VEGF is in contrast to the findings previously reported under serum-containing conditions which could be due the presence of serum in the culture milieu. These novel insights into the arterial–venous specification under serum-free conditions could provide clues for developing clinically competent vascular cells in the future. However, in the current methodology, the hESCs cultured in mTeSR1 over Matrigel were used. Matrigel is of murine origin and mTeSR1 contains certain components of xenogeneic origin. Secondly, both H1 and H9 hESCs used in the current study were originally derived under xenogeneic conditions. Matrigel-based feeder-free culture systems have been successfully replaced with the use of xeno-free matrices and culture media [54–59]. Hence, future studies using hESCs and iPSCs derived and cultured under xeno-free conditions are warranted for derivation of clinically competent ECs.

From a drug discovery and clinical therapeutic standpoint, we reduced the use of xenogeneic products and further demonstrated the safety and the ability of hESC-derived arterial and venous ECs to form microvessels upon transplantation in a mouse model. Microvessels derived from arterial and venous endothelial phenotypes were functionally integrated with the host vasculature. Furthermore, the hESC-derived arterial and venous ECs maintained the expression of the arterial marker (Ephrin-B2) and venous marker (Eph-B4), respectively. However, a few microvessels formed by arterial ECs expressed the venous marker and vice versa, which might indicate either the ECs were not sufficiently pure enough or they underwent a phenotypic change in vivo—both possibilities are equally plausible. Flow cytometry analysis of the arterial and venous markers among the ECs show that a small proportion of venous ECs express arterial markers de novo, and the proportion of these venous ECs expressing arterial markers increases upon culture under arterial conditions (and vice versa). From a phenotypic stability standpoint, ECs (especially arterial ECs) require the support of mural cells such as pericytes and vascular smooth muscle cells [3]. In the present study, only ECs (arterial or venous) were transplanted and were harvested after 2 weeks; thus, investigating the effect of absence or inclusion of supporting mural cells on the phenotypic stability/modulation would be interesting. Also, the phenotypic stability/modulation of these microvessels after long-term integration in the in vivo milieu would be of interest to understand the arterial–venous specification and modulation. Furthermore, it needs to be determined whether the enrichment of arterial or venous subtypes favors therapeutic benefit over a mixed population of ECs. It might also be plausible that both cells types have specific indications depending on the therapeutic site or diseased state as it is well known that endothelial phenotypes contribute to site-specific predisposition and/or occurrence of vascular diseases including atherosclerosis, vascular calcifications, thrombosis and aneurysms. Furthermore, the role of these endothelial subtypes in tissue engineering of vascular stents (specifically arterial or venous stents) would be exciting. These investigations would be interesting and warranted, but are beyond the scope of this study.

## Conclusion

**Fig. 8:**
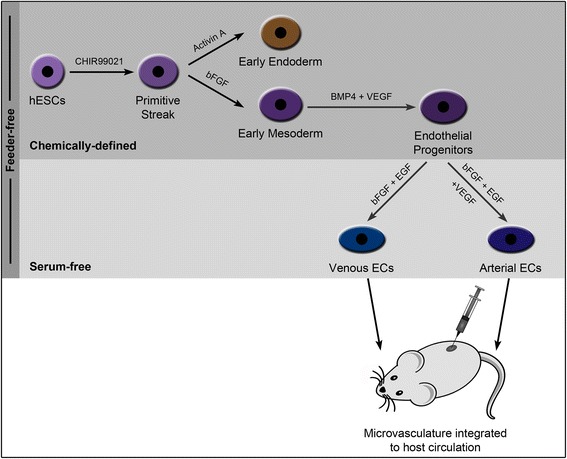
Schematic diagram summarizing the protocol for differentiation of hESCs to endothelial phenotypes. Schematic representation depicts the differentiation of hESCs to endothelial progenitors and further towards venous and arterial endothelial phenotypes that upon transplantation form functional microvessels in vivo. *bFGF* Basic fibroblast growth factor, *BMP4* Bone morphogenetic protein 4, *EC* Endothelial cells, *EGF* Epidermal growth factor, *hESC* Human embryonic stem cells, *VEGF* Vascular endothelial growth factor

In conclusion, we demonstrate the efficient differentiation of hESCs to CD34^+^CD31^+^ endothelial progenitors under feeder-free and chemically defined microenvironment by recapitulating the early embryonic vasculogenesis through the sequential intervention of GSK-3, bFGF, BMP4 and VEGF signaling pathways that results in successive emergence of PS, early mesoderm/lateral plate mesoderm, and endothelial progenitors (Fig. 8). Under feeder- and serum-free conditions, these hESC-derived endothelial progenitors could be directed to arterial and venous ECs depending on the presence or absence of VEGF. Furthermore, these hESC-derived arterial and venous ECs demonstrate differences at the transcriptome and secretome levels, and form functional microvessels that integrate with the host circulation and maintain their respective phenotypes in vivo. The efficient generation of these hESC-derived endothelial progenitors and endothelial phenotypes may be more amenable for large-scale production and expansion. Furthermore, we believe that the findings of our study could be translated using other hESCs and iPSCs for developing personalized cell therapy, drug discovery and disease-modeling applications. Furthermore, the access to robust differentiation of human PSCs to arterial and venous ECs under defined conditions could provide a potential human model to study arterial–venous specification for various basic and pharmaceutical research applications and in the future towards clinical regenerative therapies.

## Additional file

Additional file 1: Figure S1. Immunocytochemical analysis of pluripotency status of hESCs cultured over Matrigel. **Figure S2.** Immunocytochemical analysis of pluripotency status of hESCs cultured over fibronectin. **Figure S3.** Time-course analysis of early differentiation of hESCs towards mesodermal lineage. **Figure S4.** Time-course analysis of early differentiation of hESCs towards endodermal lineage. **Figure S5.** Differentiation of H9-hESCs towards endothelial lineage. **Figure S6.** Effect of VEGF on hESC-derived endothelial cells under serum-free conditions. **Figure S7.** Survey of angiocrine secretory profile of arterial and venous ECs using angiogenesis antibody array. **Table S1**: Legend of coordinates for the human angiogenesis array. **Table S2.** Sequences of primers used for real-time RT-PCR. **Table S3.** List of antibodies used for flow cytometry. **Table S4.** List of antibodies used for immunocytochemistry. **Supplementary Methods.** (PDF 17154 kb)

## Abbreviations

Art-EC: Arterial endothelial cells
bFGF: Basic fibroblast growth factor
BMP4: Bone morphogenetic protein 4
cAMP: Cyclic adenosine monophosphate
EC: Endothelial cells
ECM: Extracellular matrix
EGF: Epidermal growth factor
EMT: Epithelial–mesenchymal transition
ESFM: Endothelial serum-free medium
FBS: Fetal bovine serum
GSK-3: Glycogen synthase kinase-3
HCAEC: Human coronary artery endothelial cells
hESC: Human embryonic stem cells
HUVEC: Human umbilical vein endothelial cells
IGF: Insulin-like growth factor
iPSC: Induced pluripotent stem cell
PDGF: Platelet-derived growth factor
PS: Primitive streak
PSC: Pluripotent stem cells
RT-PCR: Reverse transcriptase-polymerase chain reaction
Shh: Sonic hedgehog
TGF: Transforming growth factor
VE-CAD: Vascular endothelial cadherin
VEGF: Vascular endothelial growth factor
VEGFR2: Vascular endothelial growth factor receptor-2
Ven-EC: Venous endothelial cells
vWF: von Willebrand factor
