# Supplementary material for: Efficient differentiation of human embryonic stem cells to arterial and venous endothelial cells under feeder- and serum-free conditions
Source: Stem Cell Res Ther. 2015 Dec 30;6:261. doi: 10.1186/s13287-015-0260-5 (PMC4697311; doi:10.1186/s13287-015-0260-5)
Supplement: Additional file 1: Figure S1. — Immunocytochemical analysis of pluripotency status of hESCs cultured over Matrigel. Figure S2. Immunocytochemical analysis of pluripotency status of hESCs cultured over fibronectin. Figure S3. Time-course analysis of early differentiation of hESCs towards mesodermal lineage. Figure S4. Time-course analysis of early differentiation of hESCs towards endodermal lineage. Figure S5. Differentiation of H9-hESCs towards endothelial lineage. Figure S6. Effect of VEGF on hESC-derived endothelial cells under serum-free conditions. Figure S7. Survey of angiocrine secretory profile of arterial and venous ECs using angiogenesis antibody array. Table S1: Legend of coordinates for the human angiogenesis array. Table S2. Sequences of primers used for real-time RT-PCR. Table S3. List of antibodies used for flow cytometry. Table S4. List of antibodies used for immunocytochemistry. Supplementary Methods. (PDF 17154 kb) [file 13287_2015_260_MOESM1_ESM.pdf]

Contents

1. Supplementary Figure Legends
2. Supplementary Figure S1
3. Supplementary Figure S2
4. Supplementary Figure S3
5. Supplementary Figure S4
6. Supplementary Figure S5
7. Supplementary Figure S6
8. Supplementary Figure S7
9. Supplementary Figure S8
10. Supplementary Tables
11. Supplementary Methods

Supplementary Figure Legends

Figure S1. Immunocytochemical analysis of pluripotency status of hESCs cultured over Matrigel.

Phase contrast photomicrographs show the compact, well defined morphology of hESC colonies upon culture in mTeSR1 over Matrigel. Immunofluorescence micrographs show the expression of pluripotency markers OCT4, SSEA4, TRA-1-60, TRA-1-81 and alkaline phosphatase (AP) by H1-hESCs **(a)** and H9-hESCs **(b)**. Scale bars: 500µm.

Figure S2. Immunocytochemical analysis of pluripotency status of hESCs cultured over fibronectin.

Phase contrast photomicrographs show the compact, well defined morphology of H1-hESCs **(a)** and H9-hESCs **(d)** colonies upon culture in mTeSR1 over fibronectin for 24 hours. Real time RT-PCR comparison of expression of pluripotency genes (*OCT4, SOX2, NANOG*) by the H1-hESCs **(b)** and H9-hESCs **(e)** cultured over Matrigel and fibronectin. The plots show no significant difference in the gene expression between the cells grown on Matrigel and fibronectin. For gene expression plots, the levels of expression were normalized to corresponding *β-ACTIN* values and are shown as relative to that of respective undifferentiated hESCs cultured over Matrigel. Immunofluorescence micrographs show the expression of pluripotency markers OCT4, SSEA4, TRA-1-60, TRA-1-81and alkaline phosphatase (AP) by H1-hESCs **(c)** and H9-hESCs **(f)**. Scale bars: 200µm.

Figure S3. Time course analysis of early differentiation of hESCs towards mesodermal lineage.

(a) Schematic representation of differentiation of hESCs towards mesoderm using bFGF (Gi.F.F) after 24 hours of treatment with CHIR99021 (+GSKi). Condition without bFGF (Gi.-.-) and the one with bFGF+FGF receptor inhibitor PD173074 (Gi.FFi.FFi) were used as controls. **(b)** Representative photomicrographs display the change in the morphology of the hESC colonies under different differentiation conditions after 2 and 3 days of differentiation. The cells can be seen spreading and migrating away from the center of the colony similar to early migratory events observed in early embryonic development. **(c)** mRNA expression kinetics of markers associated with PS

(mesendoderm), anterior PS/ endoderm, mesodermal subsets and epithelial-mesenchymal transition (EMT) after differentiation of hESCs under the three different conditions. For all gene expression plots, the levels of expression were log normalized to corresponding *β-ACTIN* values and are shown as relative to that of undifferentiated hESCs. Scale bars: 500μm. Error bars: s.d. ($n \geq 3$). *$p<0.05$.**$p<0.01$.p values represent the levels of significance in relation to (Gi.-.-).

Figure S4. Time course analysis of early differentiation of hESCs towards endodermal lineage.

(a) Schematic representation of differentiation of hESCs towards endoderm using Activin A (Gi.A.A) after 24 hours of treatment with CHIR99021 (+GSKi). Condition without Activin A (Gi.-.-) and the one with Activin+TGFβ inhibitor SB431542 (Gi.ASb.ASb) were used as controls. **(b)** Representative photomicrographs display the change in the morphology of the hESC colonies under different differentiation conditions after 2 and 3 days of differentiation. The cells can be seen spreading and migrating away from the center of the colony similar to early migratory events observed in early embryonic development. **(c)** mRNA expression kinetics of markers associated with PS (PS/mesendoderm), anterior PS / endoderm, mesodermal subsets and epithelial-mesenchymal transition (EMT) after differentiation of hESCs under the three different conditions. For all gene expression plots, the levels of expression were log normalized to corresponding *β-ACTIN* values and are shown as relative to that of undifferentiated hESCs. Scale bars: 500μm. Error bars: s.d. ($n \geq 3$). *$p<0.05$.**$p<0.01$.p values represent the levels of significance in relation to (Gi.-.-).

Figure S5. Differentiation of H9-hESCs towards endothelial lineage.

Representative flow cytometry overlays display the kinetics of co-expression of VEGFR2 and CD34 **(a)**, CD31 and CD34 **(b)** upon induction of H9-hESCs with BMP4 (Gi.F.B), VEGF (Gi.F.V) and BMP4+VEGF (Gi.F.BV) over a differentiation period of 5 days.

Figure S6. Effect of VEGF on hESC-derived endothelial cells under serum-free conditions.

(a) Representative photomicrographs of endothelial cells after 24 hour exposure to increasing concentrations of VEGF reveal the apoptosis of cells at higher VEGF concentrations. (b)

Representative flow cytometry plots displaying the Annexin V/ PI staining to quantify the apoptotic cell death with increasing concentrations of VEGF. Scale bar: 500µm.

Figure S7. Plasticity of hESC-derived arterial and venous ECs.

Ability of hESC-derived ECs to maintain the arterial and venous endothelial phenotypes was investigated by cross culturing hESC-derived arterial ECs in venous media conditions (i.e., in the absence of VEGF) and hESC-derived venous ECs in arterial media conditions by supplementing the medium with VEGF. **(a**) Representative flow cytometry overlays display the expression of arterial and venous markers among H1-Art-ECs, H1-Art-ECs cultured in the absence of VEGF [H1-Art-ECs (Venous)], H1-Ven-ECs and H1-Ven-ECs cultured in the presence of VEGF [H1-Ven-ECs (Arterial)]. **(b)** Bar charts show the percentage of H1-hESC-derived ECs (top panel) and H9-hESC-derived ECs (bottom panel) expressing arterial and venous markers upon culture in different culture conditions. The different culture conditions are represented as A (Art-ECs in arterial media), A to V (Art-ECs cultured in venous media), V (Ven-ECs in venous media) and V to A (Ven-ECs in arterial media). Error bars: s.d. ($n \geq 3$). *$p<0.05$ (paired Student t test).

Figure S8. Survey of angiocrine secretory profile of arterial and venous ECs using angiogenesis antibody array.

Graphical representation of the relative amounts of 55 angiocrine factors secreted by H1-hESC derived Ven-ECs and Art-ECs analyzed using angiogenesis antibody array. The bars represent relative amounts of factors secreted based on densitometric analysis of relative pixel density of the blots. Error bars: s.d. of 2 independent experiments. *$p<0.05$.**$p<0.01$.

Table S1: Legend of coordinates for the human angiogenesis array.

Table S2: Sequences of primers used for real time RT-PCR.

Table S3: List of antibodies used for flow cytometry.

Table S4: List of antibodies used for immunocytochemistry.

Figure S1

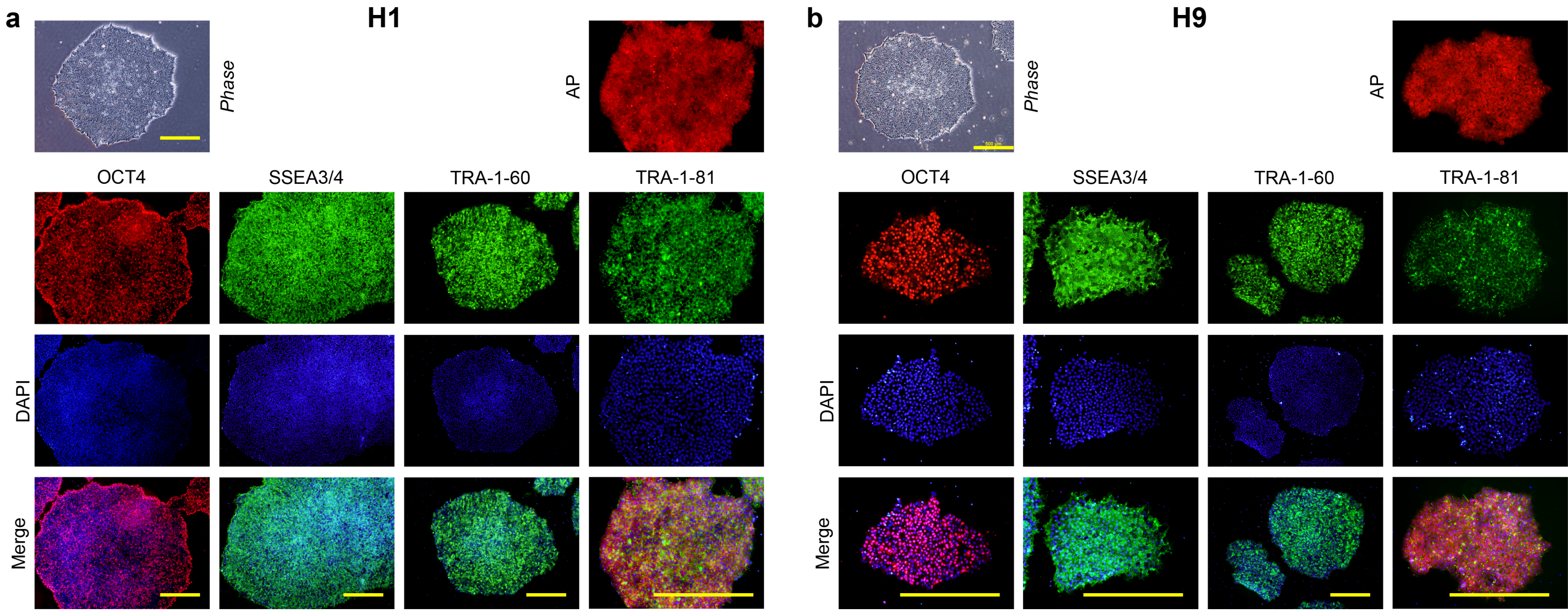
a
H1
Phase
AP
OCT4
SSEA3/4
TRA-1-60
TRA-1-81
DAPI
Merge
b
H9
Phase
AP
OCT4
SSEA3/4
TRA-1-60
TRA-1-81
DAPI
Merge

Figure S2

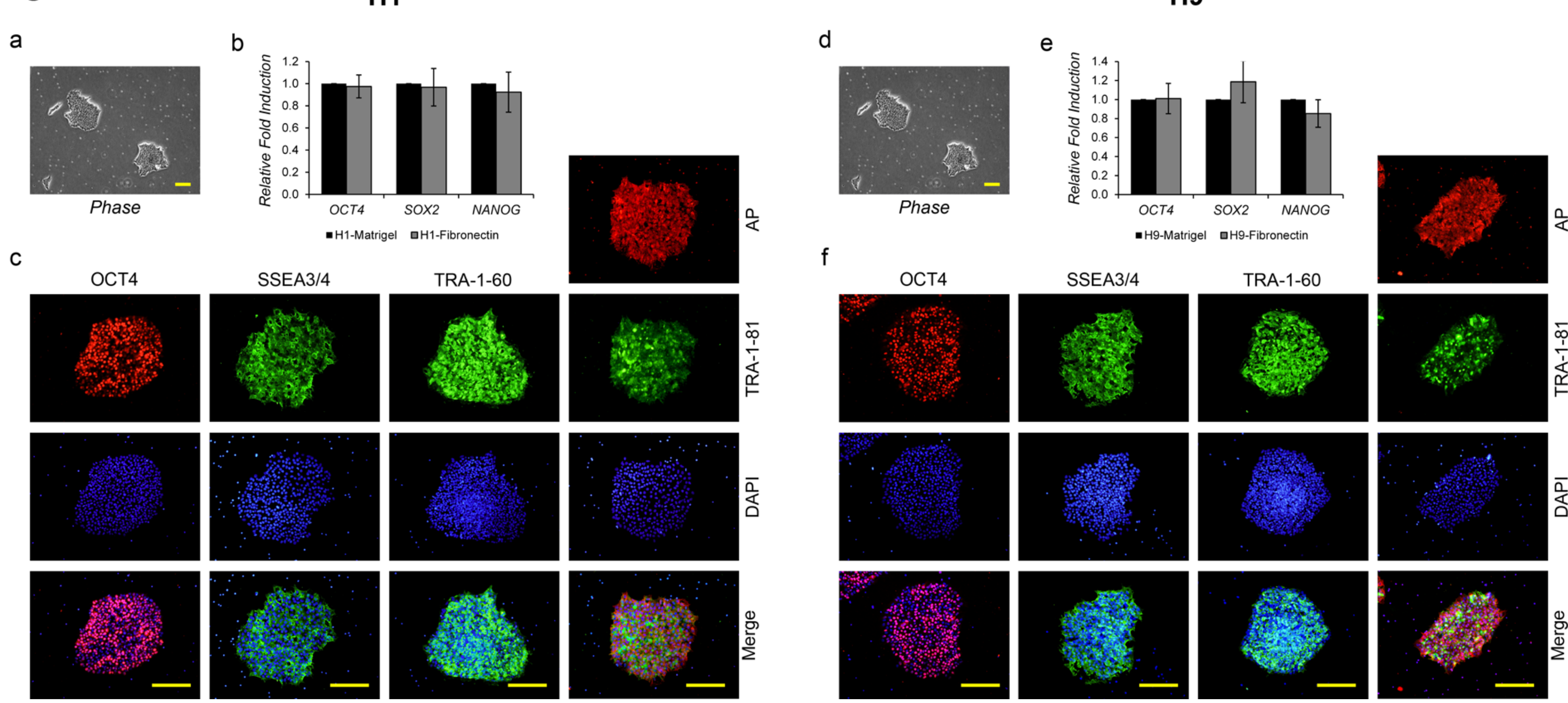

H1
H9
a
b
c
d
e
f
Phase
Relative Fold Induction
OCT4
SOX2
NANOG
H1-Matrigel
H1-Fibronectin
H9-Matrigel
H9-Fibronectin
OCT4
SSEA3/4
TRA-1-60
AP
TRA-1-81
DAPI
Merge

Figure S3

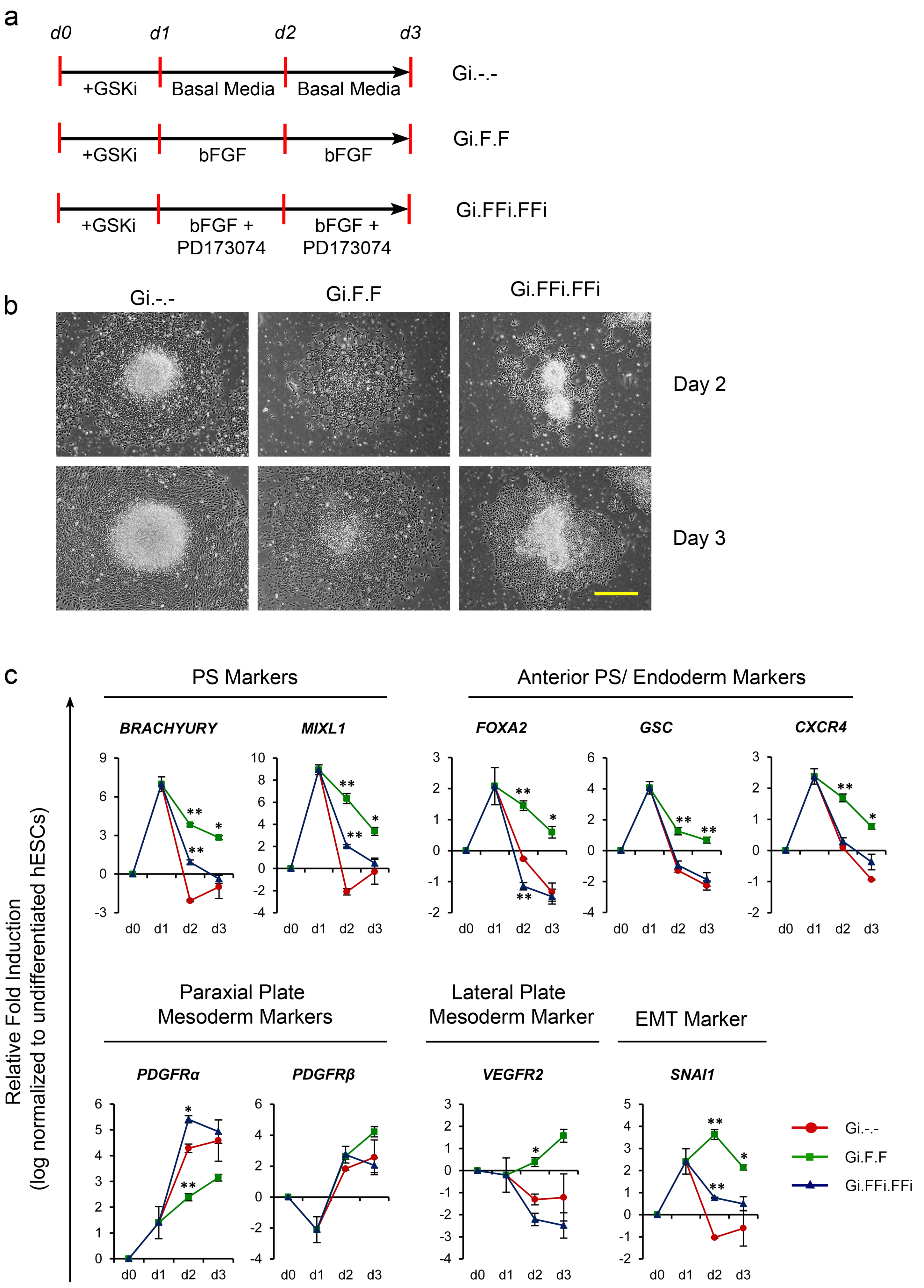
a
d0
d1
d2
d3
+GSKi
Basal Media
Basal Media
Gi.-.-
+GSKi
bFGF
bFGF
Gi.F.F
+GSKi
bFGF + PD173074
bFGF + PD173074
Gi.FFi.FFi
b
Gi.-.-
Gi.F.F
Gi.FFi.FFi
Day 2
Day 3
c
PS Markers
Anterior PS/ Endoderm Markers
BRACHYURY
MIXL1
FOXA2
GSC
CXCR4
Relative Fold Induction
(log normalized to undifferentiated hESCs)
Paraxial Plate Mesoderm Markers
Lateral Plate Mesoderm Marker
EMT Marker
PDGFRα
PDGFRβ
VEGFR2
SNAI1
d0
d1
d2
d3
Gi.-.-
Gi.F.F
Gi.FFi.FFi

Figure S4

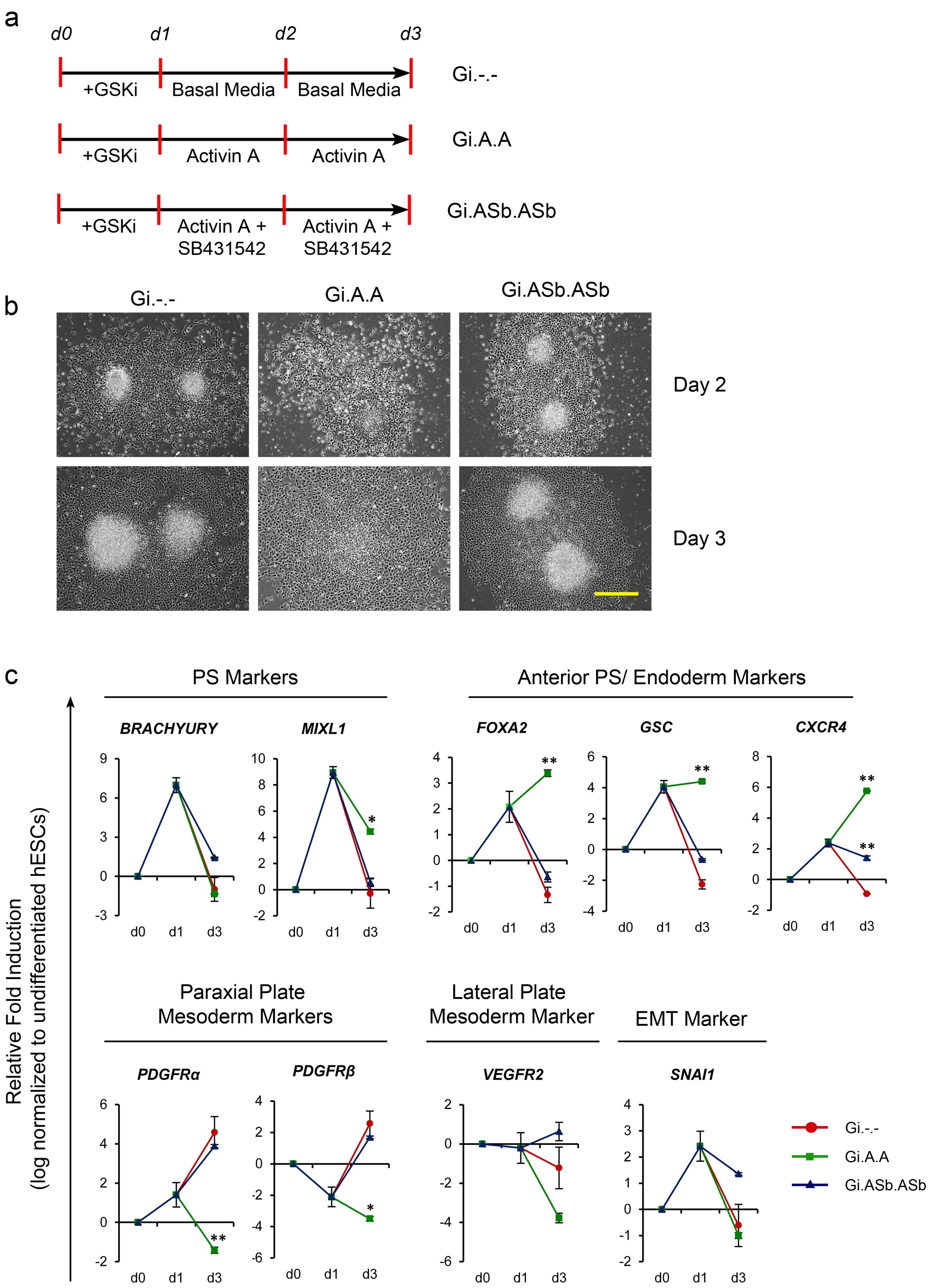
a
d0
d1
d2
d3
+GSKi
Basal Media
Basal Media
Gi.-.-
+GSKi
Activin A
Activin A
Gi.A.A
+GSKi
Activin A + SB431542
Activin A + SB431542
Gi.ASb.ASb
b
Gi.-.-
Gi.A.A
Gi.ASb.ASb
Day 2
Day 3
c
PS Markers
Anterior PS/ Endoderm Markers
BRACHYURY
MIXL1
FOXA2
GSC
CXCR4
Relative Fold Induction
(log normalized to undifferentiated hESCs)
Paraxial Plate Mesoderm Markers
Lateral Plate Mesoderm Marker
EMT Marker
PDGFRα
PDGFRβ
VEGFR2
SNAI1
d0
d1
d3
Gi.-.-
Gi.A.A
Gi.ASb.ASb

Figure S5

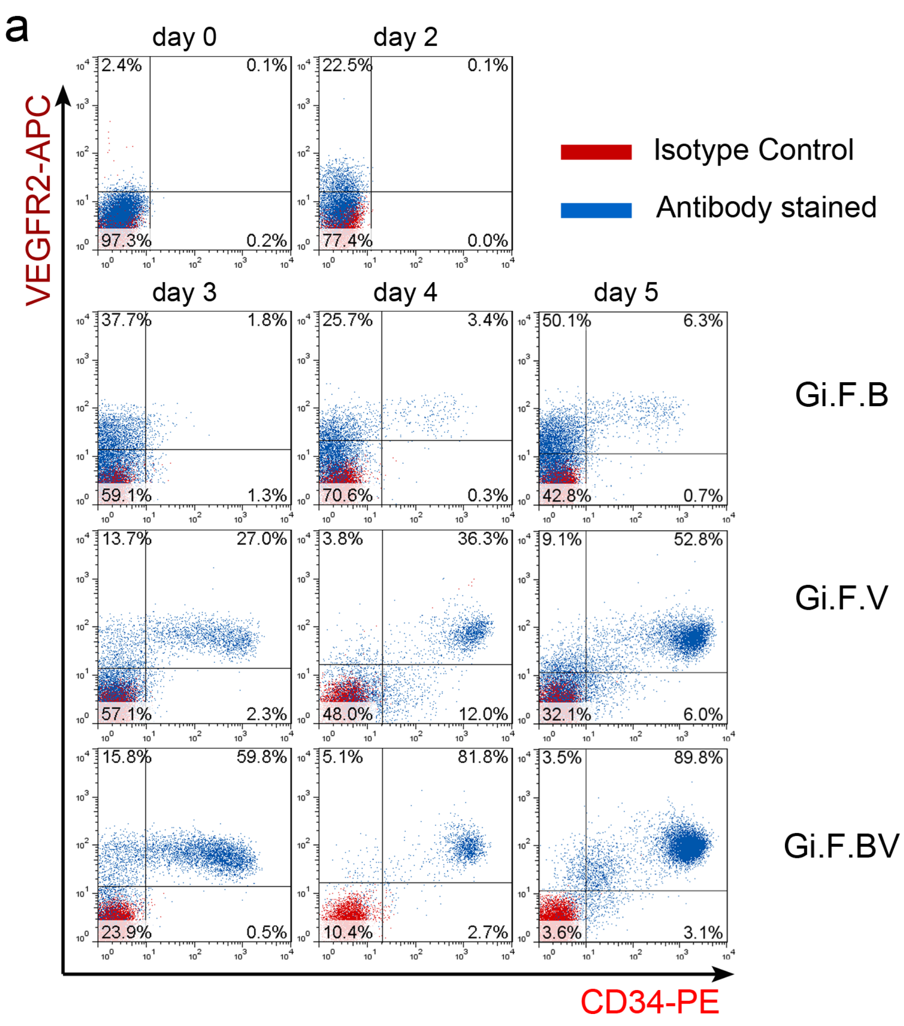
a
b
day 0
day 2
day 3
day 4
day 5
VEGFR2-APC
CD31-APC
CD34-PE
Isotype Control
Antibody stained
Gi.F.B
Gi.F.V
Gi.F.BV
2.4%
0.1%
97.3%
0.2%
22.5%
0.1%
77.4%
0.0%
37.7%
1.8%
59.1%
1.3%
25.7%
3.4%
70.6%
0.3%
50.1%
6.3%
42.8%
0.7%
13.7%
27.0%
57.1%
2.3%
3.8%
36.3%
48.0%
12.0%
9.1%
52.8%
32.1%
6.0%
15.8%
59.8%
23.9%
0.5%
5.1%
81.8%
10.4%
2.7%
3.5%
89.8%
3.6%
3.1%
0.5%
0.0%
99.4%
0.0%
0.5%
0.0%
99.3%
0.3%
5.0%
0.5%
92.0%
2.6%
4.8%
4.4%
90.4%
0.4%
6.2%
4.1%
87.9%
1.8%
6.5%
24.2%
62.9%
6.4%
8.4%
48.2%
41.1%
2.3%
6.3%
56.0%
34.5%
3.2%
4.0%
48.1%
36.8%
11.1%
1.1%
81.6%
15.0%
2.3%
0.9%
90.5%
6.0%
2.5%

Figure S6

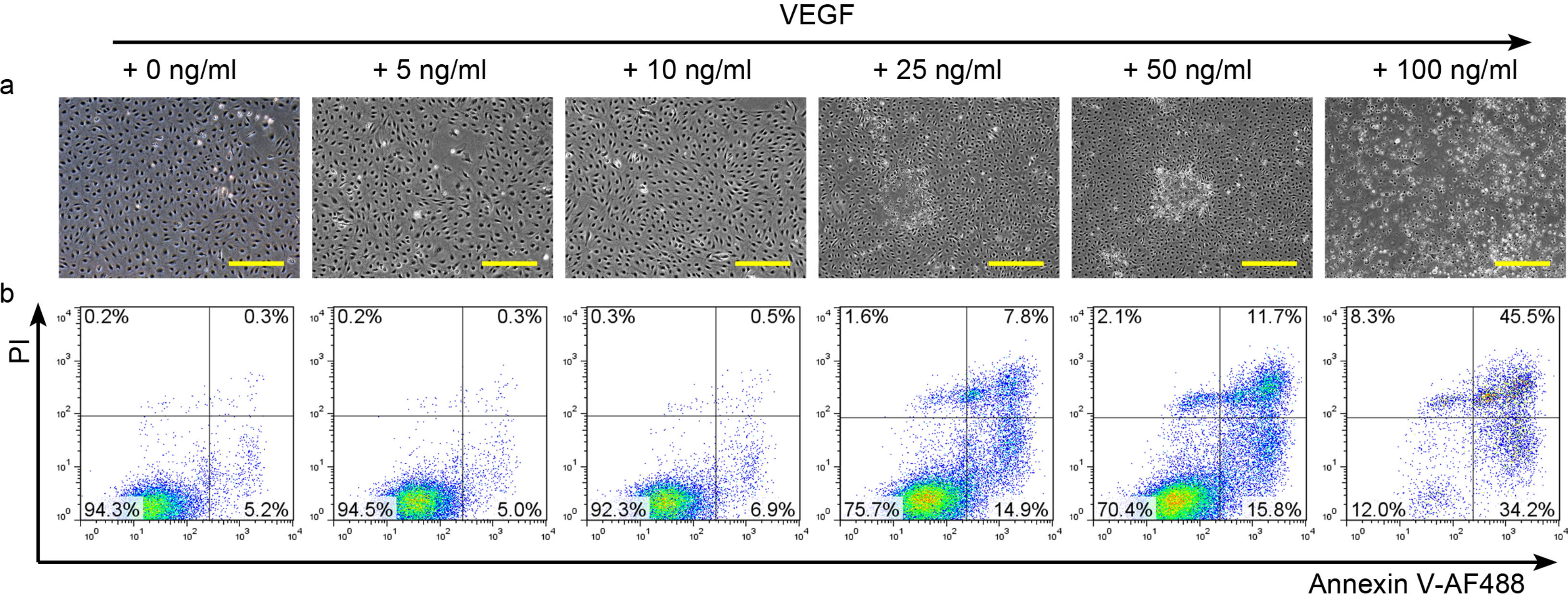
VEGF
+ 0 ng/ml
+ 5 ng/ml
+ 10 ng/ml
+ 25 ng/ml
+ 50 ng/ml
+ 100 ng/ml
a
b
PI
0.2%
0.3%
94.3%
5.2%
0.2%
0.3%
94.5%
5.0%
0.3%
0.5%
92.3%
6.9%
1.6%
7.8%
75.7%
14.9%
2.1%
11.7%
70.4%
15.8%
8.3%
45.5%
12.0%
34.2%
Annexin V-AF488

Figure S7

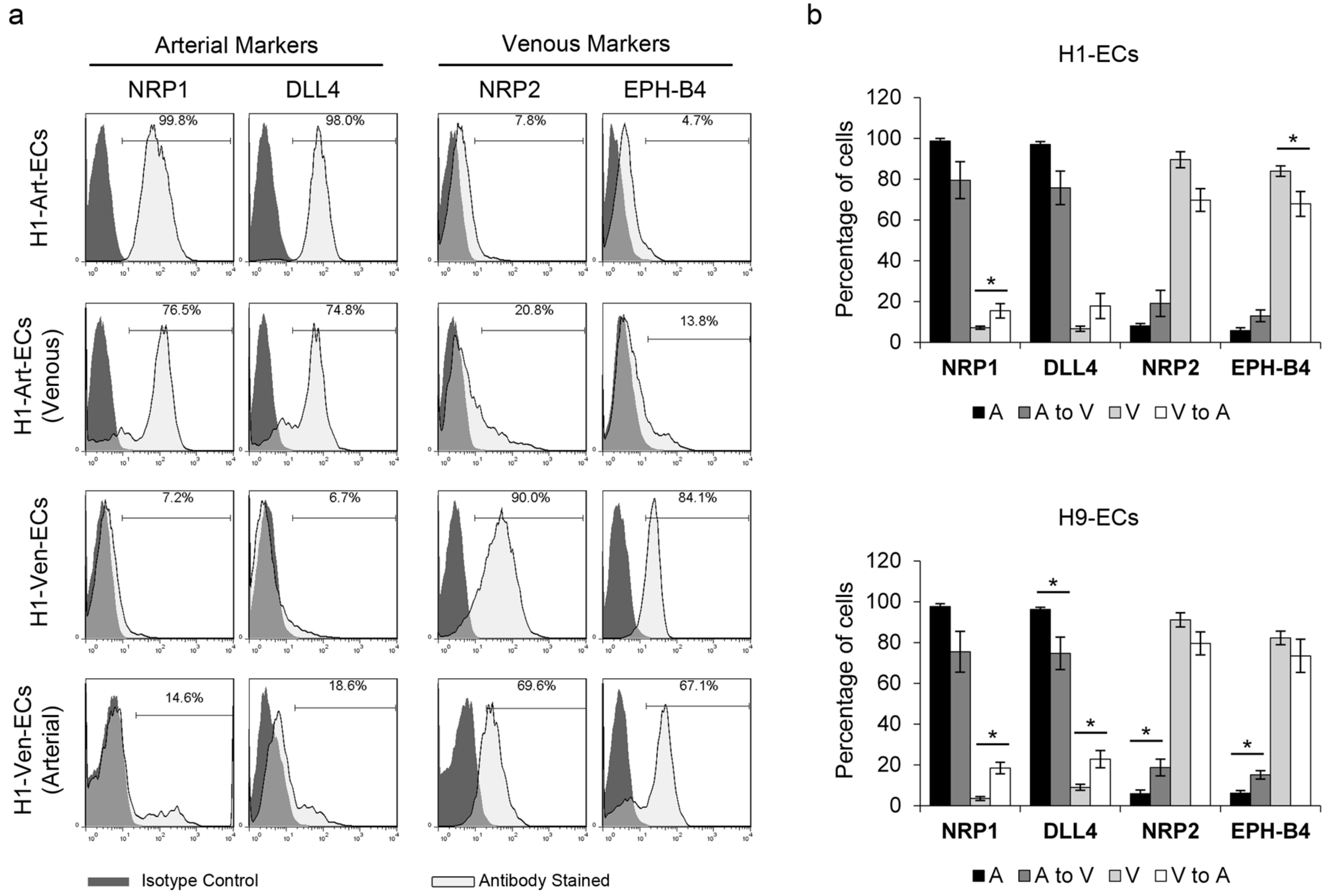

a
Arterial Markers
Venous Markers
NRP1
DLL4
NRP2
EPH-B4
H1-Art-ECs
99.8%
98.0%
7.8%
4.7%
H1-Art-ECs (Venous)
76.5%
74.8%
20.8%
13.8%
H1-Ven-ECs
7.2%
6.7%
90.0%
84.1%
H1-Ven-ECs (Arterial)
14.6%
18.6%
69.6%
67.1%
Isotype Control
Antibody Stained
b
H1-ECs
Percentage of cells
120
100
80
60
40
20
0
NRP1
DLL4
NRP2
EPH-B4
A
A to V
V
V to A
H9-ECs
Percentage of cells
NRP1
DLL4
NRP2
EPH-B4
A
A to V
V
V to A

Figure S8

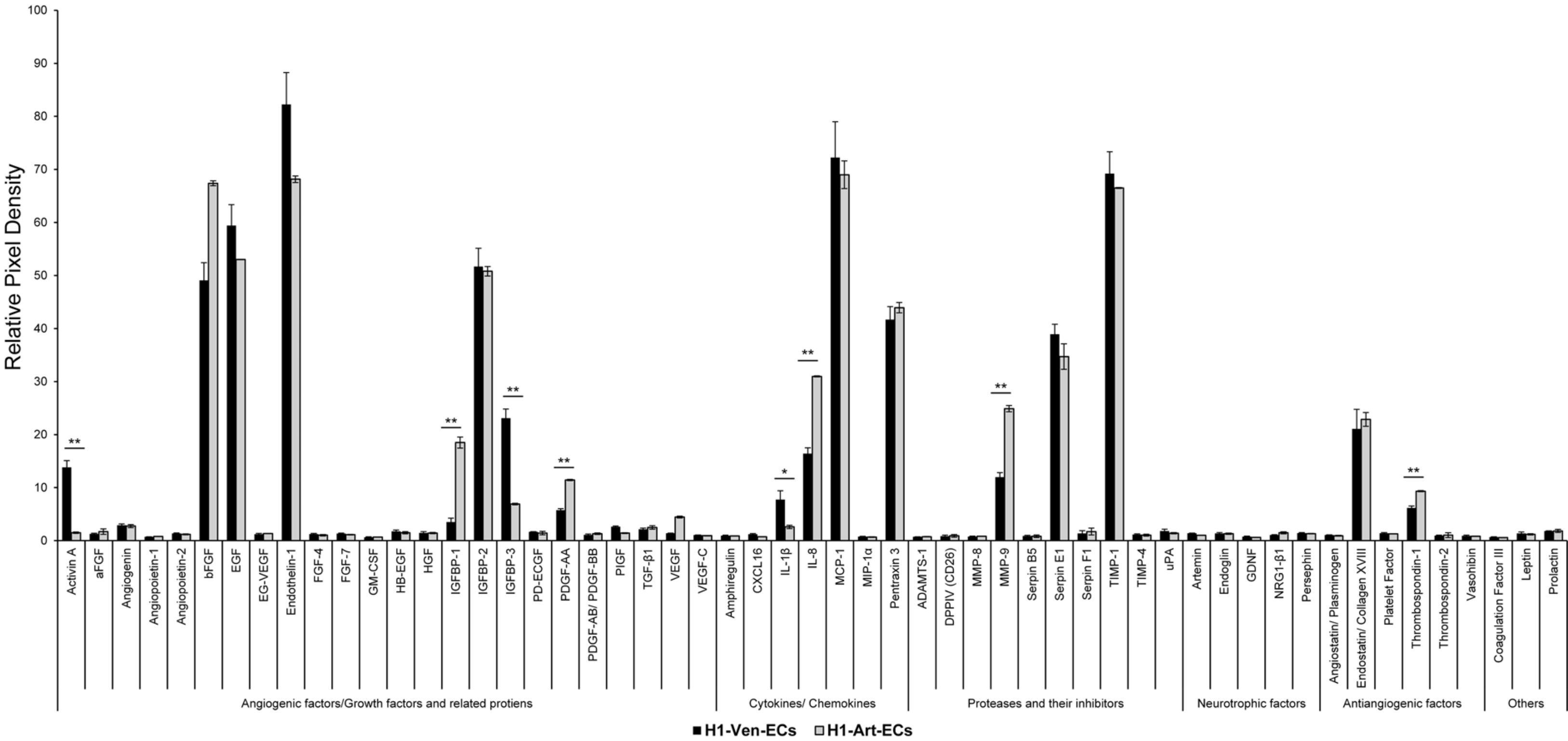
Relative Pixel Density
Activin A
aFGF
Angiogenin
Angiopoietin-1
Angiopoietin-2
bFGF
EGF
EG-VEGF
Endothelin-1
FGF-4
FGF-7
GM-CSF
HB-EGF
HGF
IGFBP-1
IGFBP-2
IGFBP-3
PD-ECGF
PDGF-AA
PDGF-AB/ PDGF-BB
PlGF
TGF-β1
VEGF
VEGF-C
Amphiregulin
CXCL16
IL-1β
IL-8
MCP-1
MIP-1α
Pentraxin 3
ADAMTS-1
DPPIV (CD26)
MMP-8
MMP-9
Serpin B5
Serpin E1
Serpin F1
TIMP-1
TIMP-4
uPA
Artemin
Endoglin
GDNF
NRG1-β1
Persephin
Angiostatin/ Plasminogen
Endostatin/ Collagen XVIII
Platelet Factor
Thrombospondin-1
Thrombospondin-2
Vasohibin
Coagulation Factor III
Leptin
Prolactin
Angiogenic factors/Growth factors and related protiens
Cytokines/ Chemokines
Proteases and their inhibitors
Neurotrophic factors
Antiangiogenic factors
Others
H1-Ven-ECs
H1-Art-ECs

SUPPLEMENTARY TABLES

Table S1: Legend of coordinates for the human angiogenesis array.

The location of controls and captured antibodies in the Human Angiogenesis Array is listed below. The picture shows the coordinates of each control/ captured antibody in the array.

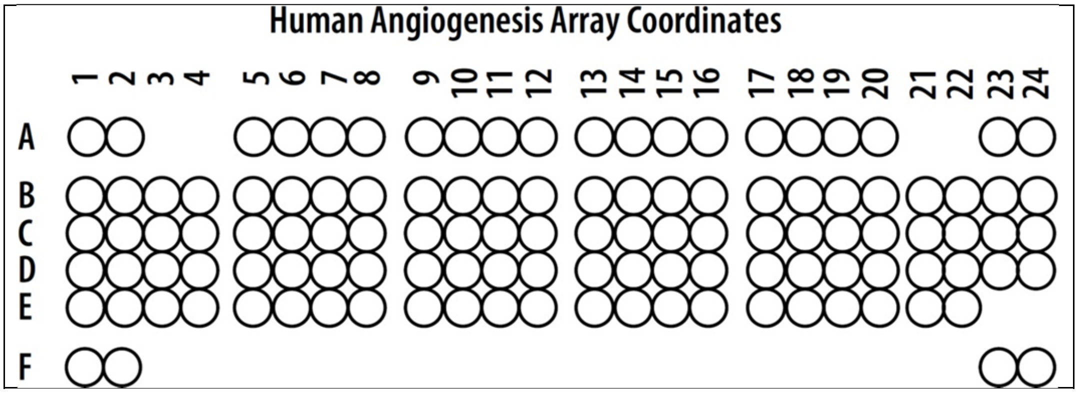

Coordinates	Target/ Control	Alternative Name/ Abbreviation	Description
A1, A2	Reference Spots		
A5, A6	Activin-A		
A7, A8	ADAMTS-1		A Disintegrin-like and Metalloproteinase Domain with Thrombospondin Motifs 1
A9, A10	Angiogenin	ANG	
A11, A12	Angiopoietin-1	Ang-1	
A13, A14	Angiopoietin-2	Ang-2	
A15, A16	Angiostatin/Plasminogen		
A17, A18	Amphiregulin	AR	
A19, A20	Artemin		
A23, A24	Reference Spots		
B1, B2	Coagulation Factor-III	TF	
B3, B4	CXCL16		
B5, B6	DPPIV	CD26	Dipeptidyl peptidase IV
B7, B8	EGF		Epidermal Growth Factor
B9, B10	EG-VEGF	PK1	Endocrine Gland-derived Vascular Endothelial Growth Factor
B11, B12	Endoglin	CD105	
B13, B14	Endostatin/Collagen	XVIII	
B15, B16	Endothelin-1	ET-1	
B17, B18	FGF acidic	FGF-1; aFGF	
B19, B20	FGF basic	FGF-2; bFGF	
B21, B22	FGF-4		
B23, B24	FGF-7	KGF	
C1, C2	GDNF		Glial Cell line-derived Growth Factor
C3, C4	GM-CSF		Granulocyte Macrophage Growth Factor

Coordinates	Target/ Control	Alternative Name/ Abbreviation	Description
C5, C5	HB-EGF		Heparin Binding EGF-like Growth Factor
C7, C8	HGF		Hepatocyte Growth Factor
C9, C10	IGFBP-1		Insulin-like Growth Factor Binding Protein 1
C11, C12	IGFBP-2		Insulin-like Growth Factor Binding Protein 2
C13, C14	IGFBP-3		Insulin-like Growth Factor Binding Protein 3
C15, C16	IL-1β	IL-1F2	Interleukin-1β
C17, C18	IL-8	CXCL8	Interleukin-8
C19, C20	LAP	TGF-β1	
C21, C22	Leptin		
C23, C24	MCP-1	CCL2	monocyte chemotactic and activating factor (MCAF)
D1, D2	MIP-1α	CCL3	Macrophage Inflammatory Protein-1α
D3, D4	MMP-8		Matrix Metalloproteinase-8
D5, D6	MMP-9		Matrix Metalloproteinase-9
D7, D8	NRG1-β1	HRG1-β1	Neuregulin
D9, D10	Pentraxin-3	PTX3; TSG-14	TNF-stimulated gene 14
D11, D12	PD-ECGF		Platelet-derived Endothelial Cell Growth Factor
D13, D14	PDGF-AA		Platlet-derived Growth Factor-AA
D15, D16	PDGF-AB/PDGF-BB		Platlet-derived Growth Factor-AB/BB
D17, D18	Persephin		
D19, D20	Platelet Factor-4	PF4; CXCL4	
D21, D22	PlGF		Placenta-derived Growth Factor
D23,D24	Prolactin		
E1, E2	Serpin-B5	Maspin	
E3, E4	Serpin-E1	PAI-1	
E5, E6	Serpin-F1	PEDF	
E7, E8	TIMP-1		Tissue Inhibitor of MetalloProteinases-1
E9, E10	TIMP-4		Tissue Inhibitor of MetalloProteinases-2
E11, E12	Thrombospondin-1	TSP-1	
E13, E14	Thrombospondin-2	TSP-2	
E15, E16	uPA		Uraokinase-type Plasminogen Activator
E17, E18	Vasohibin		
E19, E20	VEGF		Vascular Endothelial Growth Factor
E21, E22	VEGF-C		Vascular Endothelial Growth Factor-C
F1, F2	Reference Spots		
F23, F24	Negative Control		

Table S2: Sequences of primers used for real time RT-PCR.

**Gene**	**Primer sequence**	**Product length**	**GenBank ID**
BACTIN	F: 5'-CCAAGGCCAACCGCGAGAAGATGAC-3'<br>R: 5'-AGGGTACATGGTGGTGCCGCCAGAC-3'	587 bp	*NM_001101.3*
BRACHYURY	F: 5'-GGGTGGCTTCTTCCTGGAAC-3'<br>R: 5'-TTGGAGAATTGTTCCGATGAG-3'	172 bp	*NM_003181.2*
CD31	F: 5'-TCTATGACCTCGCCCTCCACAAA-3'<br>R: 5'-GAACGGTGTCTTCAGGTTGGTATTTCA-3'	83 bp	*NM_000442.4*
CD34	F: 5- AAATCCTCTTCCTCTGAGGCTGGA-3'<br>R: 5- AAGAGGCAGCTGGTGATAAGGGTT-3'	216 bp	*NM_001773.2*
COUP-TFII	F: CCATAGTCCTGTTCACCTCA<br>R: GGTACTGGCTCCTAACGTATTC	111 bp	*NM_021005.3*
CXCR4	F: 5'-CGCCTGTTGGCTGCCTTA-3'<br>R: 5'-ACCCTTGCTTGATGATTTCCA-3'	74 bp	*NM_003467.2*
DLL4	F: TGCTGCTGGTGGCACTTT<br>R: CTTGTGAGGGTGCTGGTT	457 bp	*NM_019074.3*
EPH-B4	F: AGAGGCCGTACTGGGACATGAG<br>R: TCCAGCATGAGCTGGTGGAG	112 bp	*NM_004444.4*
EPHRIN-B2	F: CCCAGTGACATTATCATCCC<br>R: CATCTCCTGGACGATGTACACC	108 bp	*NM_004093.3*
FOXA2	F: 5'-CCATTGCTGTTGTTGCAGGGAAGT-3'<br>R: 5'-CACCGTGTCAAGATTGGGAATGCT-3'	196 bp	*NM_021784*
GATA2	F: 5'-AGCCGGCACCTGTTGTGCAA-3'<br>R: 5'-TGACTTCTCCTGCATGCACT-3'	244 bp	*NM_032638.4*
GSC	F: 5'-GATGCTGCCCTACATGAACGT-3'<br>R: 5'-GACAGTGCAGCTGGTTGAGAAG-3'	71 bp	*NM_173849.2*
MIXL1	F: 5'-AAGCCCCAGCTGCCTGTT-3'<br>R: 5'-CCCTCCAACCCCGTTTG-3'	63 bp	*NM_031944.1*
NANOG	F: 5'-TGATTTGTGGGCCTGAAGAAAA-3'<br>R: 5'-GAGGCATCTCAGCAGAAGACA-3'	493 bp	*NM_024865.2*
NOTCH1	F: CACGCGGATTAATTTGCATCTG<br>R: TCTTGGCATACACACTCCGAGAAC	129 bp	*NM_017617.3*
NRP1	F: CATCTCCTGGTTATCCTCATTC<br>R: GTCATACTTGCAGTCTCTGTCC	137 bp	*NM_003873.5*
NRP2	F: GCATGGCAAAAACCACAAGGTAT<br>R: TGGAGCGTGGAGCTTGTTCA	76 bp	*NM_201266.1*
OCT4	F: 5'-CGTGAAGCTGGAGAAGGAGAAGCTG-3'<br>R: 5'-AAGGGCCGCAGCTTACACATGTTC-3'	247 bp	*NM_002701.4*
PAX6	F: 5'-CTGGCTAGCGAAAAGCAACAG-3'<br>R: 5'-CCCGTTCAACATCCTTAGTTTATCA-3'	66 bp	*NM_001604*
PDGFRα	F: 5'-GATTAAGCCGGTCCCAACCT-3'<br>R: 5'-GGATCTGGCCGTGGGTTT-3'	65 bp	*NM_006206*
PDGFRβ	F: 5'-TGGCAGAAGAAGCCACGTT-3'<br>R: 5'-GGCCGTCAGAGCTCACAGA-3'	63 bp	*NM_002609*
SNAI1	F: CCCACATCCTTCTCACTGC<br>R: GTCAGCCTTTGTCCTGTAGC	265 bp	*NM_005985*
SOX1	F: 5'-GCGGTAACAACTACAAAAAACTTGTAA-3'<br>R: 5'-GCGGAGCTCGTCGCATT-3'	76 bp	*NM_005986*
SOX2	F: 5'-CCGCATGTACAACATGATGG-3'<br>R: 5'-CTTCTTCATGAGCGTCTTGG-3'	370 bp	*NM_003106.2*
VE-CAD	F: 5'-AGCCCAAAGTGTGTGAGAACGC-3<br>R: 5'-CTGAGATGACCACGGGTAGGAA-3	225 bp	*NM_001795.3*
VEGFR2	F: 5-CGGCTCTTTCGCTTACTGTT-3'<br>R: 5-TCCTGTATGGAGGAGGAGGA-3'	537 bp	*NM_002253.2*
VWF	F: 5'-TTCCAGAATGGCAAGAGAGTG-3'<br>R: 5'-TGAGTTGGCAAAGTCATAAGG-3'	345 bp	*NM_000552.3*

Table S3: List of antibodies used for flow cytometry.

Protein	Antibody details	Clone	Catalogue	Supplier
CD31-APC	Anti-Human; Mouse monoclonal IgG1	AC128	130-092-652	Miltenyi Biotec
CD34-PE	Anti-Human; Mouse monoclonal IgG1	4H11	12-0349	eBioscience
CXCR4-PE	Anti-Human; Mouse monoclonal IgG2a	12G5	561733	BD Pharmingen
DLL4-PE	Delta-like protein-4<br>Anti-Human; Mouse monoclonal IgG1	MHD4-46	346505	Biolegend
EphB4-FITC	Ephrin Receptor B4<br>Anti-Human; Rat monoclonal IgG1	395810	FAB3038F	R&D Systems
NRP1-APC	Neuropilin-1 (CD304)<br>Anti-Human; Mouse monoclonal IgG2a	12C2	354505	Biolegend
NRP2-APC	Neuropilin-2<br>Anti-Human/mouse; Mouse monoclonal IgG2A	257103	FAB22151A	R&D Systems
VE-Cadherin-APC	Vascular Endothelial Cadherin<br>Anti-Human; Mouse monoclonal IgG2B	123413	FAB9381A	R&D Systems
VEGFR2-APC	Anti-Human; Mouse monoclonal IgG1	ES8-20E6	130-093-601	Miltenyi Biotec
FcR Blocking Reagent	Human FcγR blocking reagent	-	130-059-901	Miltenyi Biotec
Isotype control-AF488	Alexa Flour 488 isotype control<br>Anti-Human; mouse monoclonal IgG2A	G155-178	557703	BD Pharmingen
Isotype control-PE	Phycoerythrin isotype control<br>Anti-Human; mouse monoclonal IgG1	MOPC-21	559320	BD Pharmingen
Isotype control-APC	Allophycocyanin isotype control<br>Anti-Human; mouse monoclonal IgG1	X40	340442	BD Pharmingen

Table S4: List of antibodies used for immunocytochemistry.

Protein	Antibody details	Catalogue	Clone	Supplier
AP (Alkaline Phosphatase)	Anti-Human; Rabbit polyclonal	SC-30203	H-300	Santa Cruz Biotechnology
BRACHYURY	Anti-Human; Rabbit polyclonal IgG	AB20680	605502	Abcam
CD31	Anti-Human; Mouse monoclonal IgG1	BBA7	9G11	R&D Systems
OCT4	Anti-Human; Mouse monoclonal IgG	SC-5279	C-10	Santa Cruz Biotechnology
OCT4	Anti-Human; Rabbit polyclonal IgG	AB19857	-	Abcam
SSEA4	Anti-Human; Mouse monoclonal IgG3	MAB4304	MC-813-70	Millipore (Chemicon)
TRA-1-60	Anti-Human; Mouse monoclonal	MAB4360	TRA-1-60	Millipore (Chemicon)
TRA-1-81	Anti-Human; Mouse monoclonal	MAB4381	TRA-1-81	Millipore (Chemicon)
VE-CADHERIN	Anti-Human; Mouse monoclonal IgG2B	MAB9381	123413	R&D Systems
vWF	Anti-Human; Rabbit polyclonal IgG	GTX26994	-	GeneTex
Secondary antibody	Alexa Fluor 488<br>Goat anti-mouse IgG (H+L)	A-11001	-	Molecular Probes
Secondary antibody	Alexa Fluor 594<br>Goat anti-rabbit IgG (H+L)	A-11037	-	Molecular Probes
Secondary antibody	Alexa Fluor 488<br>Goat anti-rabbit IgG (H+L)	A-11008	-	Molecular Probes
Secondary antibody	Alexa Fluor 594<br>Goat anti-mouse IgG (H+L)	A-11005	-	Molecular Probes

SUPPLEMENTARY METHODS

Culture of primary endothelial cells

Human umbilical vein endothelial cells (HUVECs, Lonza) (kind gift from Koon Gee Neoh, National University of Singapore) were cultured in Clonetics™ EGM™-2 media (endothelial growth media with 2% serum and other growth factor supplements) as per manufacturer's instructions. Human coronary artery endothelial cells (HCAECs) (Promocell) were cultured in Promocell™ ECGM-2 media (endothelial cell growth media with 2% serum and other growth factor supplements) as per manufacturer's instructions. Passage 3-5 of HUVECs and HCAECs were used for the experiments.

RNA extraction and real-time PCR

Total cellular RNA was isolated from harvested cells using RNeasyPlus Mini kit (Qiagen) and reverse transcribed using iScript™ cDNA synthesis kit (BioRad) according to manufacturer's instructions. Real-time PCR was performed in triplicates using Fast SYBR Green PCR master mix (Applied Biosystems) and Stepone Plus real-time PCR system (Applied Biosystems) as per manufacturer's instructions. Real-time PCR reaction mixtures were denatured at 94°C for 20s and cycled for 40 cylces at 95°C for 3s, 60°C for 30s, followed by melt curve stage. The expression levels of specific genes were quantified by normalization against corresponding internal control gene *β-ACTIN* and expressed as the fold change relative to control sample (undifferentiated hESCs). Details of related primer sequences used in this study are presented in **Supporting Information, Table S2**. The results are presented as mean ± standard deviations of atleast three independent experiments.

Flow Cytomerty analysis and sorting

hESCs and differentiated cells were harvested using accutase, resuspended in FACS buffer (1xPBS/ 0.5% BSA) and incubated with FcR blocking agent (1:10; MiltenyiBiotec) for 10 minutes at 4°C to block non-specific binding of antibodies. For labeling of cell surface antigens, the cells were incubated with the antibodies for 10 minutes at 4°C. The list of antibodies used is presented in **Supporting Information, Table S3**. After labeling with appropriate antibodies, the cells were washed thrice with FACS buffer to remove unbound antibodies and resuspended in FACS buffer for analysis and/or sorting. The labeled cells were analyzed for surface-marker expression using Dako Cytomation CyAn ADP and sorted using a Dako Cytomation MoFlo high speed flow cytometer. The flow cytometry data was further analyzed using FlowJo v7.6.5 (TreeStar).

Immunocytochemistry

hESCs and differentiated cells were fixed with 4% paraformaldehyde (Sigma) for 20 minutes at room temperature. The fixed cells were permeabilized using PBS/ 0.1% Triton X-100 (Sigma) for 10 minutes, washed thrice with PBS/0.05% Tween-20 (Sigma) and blocked with PBS/5% goat serum for 60 minutes to block non-specific binding. Subsequently, the cells were labeled with appropriate primary antibodies (listed in **Supporting Information, Table S4**) at 4°C overnight and fluorescently labeled using appropriate secondary antibodies (listed in **Supporting Information, Table S4**) for 60 minutes. For nuclear labeling, the cells were washed and labeled with 4`,6-diamidino-2-phenylindole (DAPI; Sigma) for 3 minutes. After washing thrice, the cells were observed using fluorescence microscope (Olympus IX70). The images in Figure 1 were obtained using laser scanning confocal microscope, while all other fluorescent images were obtained using epifluorescence microscope.

Acetylated-low density lipoprotein uptake assay

To demonstrate the ability of ECs to phagocytize low-density lipoprotein (LDL), hESC-derived arterial and venous ECs, were incubated with 10µg/ml of Dil-acetylated-LDL (Dil-Ac-LDL; Molecular Probes) for 4h. The cells were washed with PBS and the nuclei counterstained with Hoescht 33258 (Sigma) and observed using fluorescence microscope (Olympus IX70).

Matrigel tube formation assay

Ability of the endothelial cells to form vascular tube-like structures over Matrigel™ was analyzed as previously described [1]. Briefly, $7.5x10^{3}$ ECs were seeded onto each well of ibidi µ-angiogenesis slides that is coated with 10µl of Matrigel. After incubation for 18h, the vascular tube-like structures were labeled with 1µM Calcein-AM (Sigma) as per manufacturer’s instructions and visualized using fluorescence microscope (Olympus IX70).

Annexin V-Propidium Iodide (PI) apoptosis assay

Analysis of live cells and apoptotic cells were performed using Alexa Fluor® 488 Annexin V/Dead Cell Apoptosis Kit (Molecular Probes). Briefly, the after 24 hour exposure to varying concentration of VEGF, the cells in the culture supernatant and culture plate were collected, washed and resuspended in 1x annexin-binding buffer. Then, the cells were incubated with Alexa Fluor® 488 annexin V and PI for 15 minutes at room temperature as per manufacturer’s instructions. After the incubation period, the cells were analyzed immediately using DakoCytomationCyAn ADP and FlowJo v7.6.5.

Wound closure assay

Confluent monolayers of H1-Art-ECs, H1-Ven-ECs, H9-Art-ECs, H9-Ven-ECs, HUVECs and HCAECs were scraped with a 200µl pipette tip to create a "wound" as described previously [2]. Following the creation of the wound, the cells were washed with PBS to remove debris and floating cells. H1-Art-ECs, H1-Ven-ECs, H9-Art-ECs and H9-Ven-ECs were incubated for a period of 30 hours at 37^{0}C in endothelial serum-free media supplemented with EGF (10ng/ml) and bFGF (20ng/ml). The H1-Art-ECs and H9-Art-Ecs were not supplemented with VEGF to eliminate the bias that could be created otherwise. Similarly, HUVECs and HCAECs were incubated in respective serum containing endothelial media and necessary supplements except VEGF. Photomicrographs of the same field were acquired every 3 hours starting from 0 hours to 30 hours. The closure of the wound by migration of the cells was analyzed using T-Scratch program [3], available from www.cse-lab.ethz.ch/software.html. The results are tabulated as mean ± standard deviations of three independent experiments.

Angiocrine secretome profiles of differentiated ECs

Human Angiogenesis Proteome Profiler™ antibody array (R&D Systems) was used to survey the levels of 55 different angiocrines secreted by H1-Art-ECs and H1-Ven-ECs using media conditioned by the respective cells. The ECs were seeded onto fibronectin-coated plates at a seeding density of $1.2x10^{4}/cm^{2}$ in their respective media. After 24 hours, the media for H1-Art-ECs and H1-Ven-ECs were changed to ESFM supplemented with EGF and bFGF only and incubated for 24 hours. After the incubation, the conditioned media were collected and stored at -80^{0}C. Total protein concentration within cell-free culture supernatants were quantified using Micro BCA™ Protein Assay Kit (Thermoscientific) as per manufacturer's instructions. Cell culture supernatant containing 200µg of protein was used for the antibody array as per manufacturer's instructions. The membranes were developed using 10 minute exposure to X-ray film. The array data was quantified by densitometry analysis using Image J (NIH, USA). The co-ordinates for the antibody arrays are presented in **Supporting Information, Table S1**.

Histological analysis

Formalin-fixed and paraffin-embedded tissues were appropriately processed and sections of 5µm were stained with haematoxylin and eosin. Immunohistochemical staining of the explants were performed using EnVision+/HRP kit (Dako) after antigen retrieval using heat treatment at 95°C for 20

minutes in Citrate buffer, pH6.0. The primary antibodies used were mouse anti-human CD31 (1:50; Clone: 9G11, R&D Systems), mouse anti-human Collagen-IV (1:50; Clone: COL-94, Sigma-Aldrich), rabbit anti-human Ephrin-B2 (1:200; polyclonal, GeneTex) and rabbit anti-human Eph-B4 (1:200; polyclonal, GeneTex).

Statistical analysis

To compare the difference between means among three groups as in Figures 1, 2, S3, and S4, the statistical significance was analyzed using one way ANOVA and Tukey's HSD posthoc analysis. Similarly, the difference in means between two groups as in Figures 5, 6, S2, S7 and S8 were analyzed using Student's t-test.

SUPPLEMENTARY REFERENCES

1. Tan JY, Sriram G, Rufaihah AJ, Neoh KG, Cao T. Efficient Derivation of Lateral Plate and Paraxial Mesoderm Subtypes from Human Embryonic Stem Cells Through GSKi-Mediated Differentiation. Stem Cells Dev. 2013;22(13):1893-906. doi:10.1089/scd.2012.0590.
2. Liang CC, Park AY, Guan JL. In vitro scratch assay: a convenient and inexpensive method for analysis of cell migration in vitro. Nat Protoc. 2007;2(2):329-33. doi:10.1038/nprot.2007.30.
3. Geback T, Schulz MM, Koumoutsakos P, Detmar M. TScratch: a novel and simple software tool for automated analysis of monolayer wound healing assays. BioTechniques. 2009;46(4):265-74. doi:10.2144/000113083.
